# A Perfect Storm of Pollutants: Environmental Mixtures in the Pathogenesis of Atherosclerosis

**DOI:** 10.3390/ijms27177624

**Published:** 2026-08-25

**Authors:** Francesca Gorini, Mariangela Palazzo, Ludovica Simonini, Alessandro Tonacci, Antonio Rizza, Haotian Wu, Fabrizio Minichilli, Andrea Borghini

**Affiliations:** 1 Institute of Clinical Physiology, National Research Council, 56124 Pisa, Italy; mariangelapalazzo@cnr.it (M.P.); ludovicasimonini@cnr.it (L.S.); alessandro.tonacci@cnr.it (A.T.); fabrizio.minichilli@cnr.it (F.M.); 2 U.O.C. Cardiologia Diagnostica e Interventistica, Fondazione Toscana Gabriele Monasterio, 54100 Massa, Italy; antonio.rizza@ftgm.it; 3 Department of Environmental Health Sciences, Columbia University Mailman School of Public Health, New York, NY 10032, USA; hw2694@cumc.columbia.edu

**Keywords:** atherosclerosis, environmental mixtures, co-exposure, multipollutant exposure, oxidative stress, inflammation, lipid dysregulation, endothelial dysfunction, epigenetics

## Abstract

Atherosclerosis is a complex, multifactorial disease and the leading underlying cause of cardiovascular morbidity and mortality worldwide. Beyond traditional risk factors, growing evidence highlights the critical role of environmental exposures in modulating atherogenesis. In real-world scenarios, individuals are exposed to complex mixtures of contaminants, including particulate matter, toxic metals, pesticides, polycyclic aromatic hydrocarbons, and endocrine-disrupting chemicals. These combined exposures may exert additive, synergistic or antagonistic effects, resulting in biological responses that cannot always be predicted from single-agent exposures. At the cellular and molecular level, both chemical mixtures and environmental co-exposures converge on key pathogenic pathways, including oxidative stress, endothelial dysfunction, chronic inflammation, and lipid dysregulation. These processes may favor monocyte recruitment, foam cell formation, vascular smooth muscle cell remodeling, and plaque instability. Moreover, cumulative exposures are associated with epigenetic alterations, such as dysregulation of DNA methylation and non-coding expression patterns, which may influence long-term susceptibility to atherosclerosis. This structured narrative review synthesizes evidence from 37 studies investigating the cellular and molecular mechanisms underlying the impact of environmental mixtures and multipollutant co-exposures on atherosclerosis. By integrating evidence from epidemiological, toxicological, experimental, bioinformatics and multi-omics studies, while recognizing the predominantly associative nature of the available human evidence, we highlight emerging mechanistic insights and discuss the relevance of mixture-based approaches for improving risk assessment and informing future preventive strategies.

## 1. Introduction

Atherosclerosis is a chronic inflammatory disease of the arterial wall characterized by lipid accumulation, endothelial dysfunction, immune cell infiltration, and progressive plaque formation, which may culminate in plaque growth, destabilization, thrombosis, and acute cardiovascular events [1]. Approximately 85% of cardiovascular disease (CVD)-related deaths are attributable to atherosclerotic cardiovascular disease (ASCVD), an umbrella term encompassing ischemic heart disease (IHD), ischemic stroke, and peripheral arterial disease [2,3]. Despite advances in prevention and treatment, IHD is projected to remain the leading cause of cardiovascular death by 2050, accounting for approximately 20 million deaths globally [2,4].

While traditional risk factors such as high systolic blood pressure, elevated fasting plasma glucose, high low-density lipoprotein (LDL) cholesterol, and increased body mass index account for a substantial proportion of ASCVD burden, increasing attention has been directed toward environmental exposures as additional determinants of CVD development and progression [5,6]. Ambient air pollution accounted for 4.2 million deaths globally in 2019, nearly 70% of which were due to CVD, mainly IHD (1.9 million) and stroke (900,000) [7]. Air pollution comprises particulate matter and a wide range of chemical constituents, including heavy metals, carbonaceous compounds, nitrates, and sulfates, present in both particulate and gaseous forms [8]. Among ambient air pollutants, PM_2.5_ (particulate matter with aerodynamic diameter ≤2.5 μm) represents one of the major contributors to the cardiovascular burden associated with air pollution [9,10]. Accumulating evidence suggests that chronic exposure to air pollutants, particularly PM_2.5_, accelerates atherosclerosis through multiple interconnected pathways, including endothelial dysfunction, vascular inflammation, oxidative stress, and epigenetic regulation of genes involved in vascular homeostasis and immune responses [8].

In addition to air pollution, numerous environmental contaminants have been increasingly recognized as contributors to atherogenesis. Toxic metals such as arsenic (As), cadmium (Cd), and lead (Pb) have been associated with endothelial dysfunction and plaque development [11,12,13], whereas pesticides, polycyclic aromatic hydrocarbons (PAHs), endocrine-disrupting chemicals (EDCs), and per- and polyfluoroalkyl substances (PFAS) have been linked to oxidative stress, inflammation, metabolic dysregulation, epigenetic modifications, and vascular injury [1,14,15,16,17]. More recently, emerging contaminants, including microplastics (MPs) and nanoplastics (NPs) have attracted attention because of their potential to induce systemic inflammatory and pro-atherogenic responses [18,19]. Collectively, these findings suggest that environmental exposures might contribute to atherosclerosis through multiple interconnected biological pathways.

However, individuals are typically exposed to complex mixtures of contaminants rather than to single agents. Such combined exposures may exert additive or synergistic effects through shared biological mechanisms [20]. Environmental stressors can interact across common molecular pathways, leading to cumulative effects that may accelerate the initiation and progression of atherosclerosis [9]. However, although numerous studies have investigated individual environmental contaminants, evidence specifically addressing environmental mixtures and co-exposures and their mechanistic contribution to atherosclerosis remains fragmented across different contaminant classes, study designs, and exposure scenarios. Therefore, there is a need for a unified, mechanism-driven framework that integrates current evidence and clarifies how combined environmental exposures contribute to atherosclerotic disease development.

Under these premises, the aim of the present narrative review is to summarize current evidence on environmental contaminant mixtures and co-exposures in relation to atherosclerosis, integrating epidemiological, experimental, and exposome studies within a framework of shared molecular and cellular mechanisms of vascular injury. We synthesize the literature to highlight convergent pathways through which co-occurring environmental exposures may promote atherogenesis, including oxidative stress, endothelial dysfunction, inflammation, lipid dysregulation, and epigenetic reprogramming.

## 2. Literature Search Strategy

This review was conducted as a structured narrative review aimed at integrating evidence on the mechanisms linking environmental mixtures and atherosclerosis across epidemiological, experimental, and mechanistic studies. Unlike reviews organized primarily by individual contaminant classes, the present work adopts a mechanism-centered perspective to identify convergent and divergent biological responses across chemically heterogeneous mixtures.

In this review, the term environmental mixture encompasses several related but not identical exposure scenarios, including simultaneous exposure to multiple chemicals, multipollutant environmental systems, co-exposure experiments, and studies using statistical methods specifically developed for mixture assessment. Because these scenarios differ in composition, timing, dose, and analytical interpretation, their findings should not be considered fully interchangeable.

A comprehensive literature search was conducted to identify studies investigating environmental contaminant mixtures and co-exposures in relation to atherosclerosis and related vascular outcomes. Searches were performed in PubMed for studies published between 1 January 2000 and 31 May 2026. Only articles published in English were included.

The search strategy combined controlled vocabulary and free-text terms related to atherosclerosis, environmental co-exposures, and mixture-based approaches. The complete search strategies, including all keywords and database-specific adaptations, are provided in Supplementary Material S1.

Records were eligible if they investigated exposure to two or more environmental contaminants assessed within the same study context and reported outcomes related to atherosclerosis or vascular disease in human, animal, or experimental models. This included both (i) studies explicitly applying mixture-based statistical approaches (e.g., weighted quantile sum regression—WQS, quantile g-computation, Bayesian kernel machine regression—BKMR) and (ii) experimental or observational co-exposure studies evaluating the biological effects of two or more contaminants within the same exposure scenario.

Studies were excluded if they were: (i) not original research (e.g., reviews, editorials, commentaries), (ii) not reporting relevant cardiovascular or vascular outcomes, (iii) not addressing environmental exposures involving at least two contaminants, (iv) lacking sufficient exposure or outcome information for interpretation, or (v) not providing mechanistic or pathophysiological evidence relevant to atherosclerosis (e.g., inflammatory, oxidative stress, endothelial, metabolic, or epigenetic pathways). In addition, the reference lists of all included articles were screened to identify further eligible studies not captured by the database searches.

Environmental mixtures were defined as exposure scenarios involving concurrent exposure to multiple environmental contaminants within a shared environmental or biological context, including chemical mixtures (e.g., co-occurring pollutants such as metals, persistent organic pollutants (POPs), PFAS, pesticides, plasticizers) and multipollutant air pollution systems.

A total of 526 records were identified through PubMed and screened by title and abstract. Thirty-seven articles were retrieved for full-text assessment and deemed eligible for inclusion in the review. For each eligible study, information was extracted on study design, population or experimental model, exposure composition and assessment, outcome or biological pathway, main findings, and relevance to atherosclerosis.

As this was a structured narrative review, no formal risk-of-bias assessment was performed. Nevertheless, study design, exposure assessment, outcome characterization, and consistency across studies were considered during evidence interpretation.

## 3. Biological Mechanisms Underlying the Atherogenic Effects of Environmental Mixtures

Despite their heterogeneity, environmental mixtures appear to converge on a limited number of biological pathways known to contribute to atherosclerosis. Available evidence highlights inflammation, oxidative stress, endothelial dysfunction, lipid dysregulation, and epigenetic alterations as key mechanisms potentially linking cumulative environmental exposures to vascular injury and atherogenesis. Because these mechanisms are highly interconnected, individual studies frequently provide evidence across multiple biological domains. Consequently, some studies are discussed in more than one subsection when they contribute to the understanding of different pathways involved in atherogenesis.

### 3.1. Inflammation

Inflammation is a central feature of atherosclerosis, driving both the initiation and progression of vascular lesions [21]. Atherosclerosis is characterized by chronic low-grade inflammation, involving endothelial activation, immune cell recruitment, and the release of pro-inflammatory cytokines and adhesion molecules [22]. These processes contribute to the formation of atherosclerotic plaque and its consequent clinical events [22].

Environmental contaminants can exacerbate these processes by activating inflammatory signaling pathways, thereby accelerating plaque formation and instability. PAHs, a class of combustion-derived organic pollutants commonly encountered through inhalation and dietary exposure, have been associated with an increased risk of IHD, angina, and MI when overall exposure is assessed through combined urinary concentrations of multiple PAH metabolites [23,24,25]. In an in vivo murine model, Rojas and colleagues investigated the effects of PAH exposure on systemic inflammation and reported significantly increased serum interleukin (IL)-6 and interferon-gamma (IFN-γ) levels in mice exposed to a mixture of phenanthrene, fluoranthene, and pyrene when compared with controls [26]. IL-6 and IFN-γ are key mediators of pro-inflammatory and immunoregulatory responses, and their increased levels are consistent with activation of nuclear factor kappa-light-chain-enhancer of activated B cells (NF-κB)-dependent inflammatory pathways involved in vascular inflammation [27,28,29]. No significant differences were observed for IL-10, IL-17A, and tumor necrosis factor alpha (TNF-α) [26]. Conversely, an in vitro experimental study reported that a mixture of 16 priority-controlled PAHs significantly increased TNF-α expression across all exposure groups [30]. Activation of the NF-κB signaling pathway was also observed at higher exposure levels, accompanied by increased expression of downstream pro-inflammatory mediators, including IL-1β, IL-6, and TNF-α, indicating induction of inflammatory signaling in vascular endothelial cells [30]. Consistent with these experimental findings, a cross-sectional National Health and Nutrition Examination Survey (NHANES) study, including participants with urinary measurements of seven monohydroxylated PAH metabolites, found a positive association between overall PAH mixture exposure and CVD prevalence [31]. This association was partially mediated by inflammatory markers, particularly the neutrophil-to-lymphocyte ratio (NLR) and the systemic immune-inflammation index, both of which have been associated with early atherosclerosis, while 2-hydroxyfluorene emerged as the major contributor to the mixture effect [31,32,33].

These findings were further supported by an NHANES-based study investigating the association between blood concentrations of trihalomethanes (THMs), disinfection by-products formed during water chlorination, and CVD risk [34,35,36]. Although evidence linking THMs to CVD remains limited, THM exposure has been associated with hypertension and type 2 diabetes, two established cardiovascular risk factors [37,38]. WQS regression revealed a significant positive association between overall THM mixture exposure and CVD risk, with NLR identified as a significant mediator of this relationship [34]. Network toxicology analyses further implicated inflammation-related genes, particularly those involved in IL-17 signaling, a pathway implicated in the pathogenesis of atherosclerosis, hypertension, coronary artery disease, and other cardiovascular conditions, as potential mechanistic links between THM exposure and CVD [34,39].

This pattern has also been observed for other classes of environmental mixtures. Using data from a large cross-sectional study, Wu et al. investigated the mechanisms underlying the combined effects of multiple pesticide exposures on ASCVD through integrated epidemiological and bioinformatics analyses [14]. Pesticides, including herbicides, insecticides, fungicides, and rodenticides, are ubiquitous and persistent environmental contaminants to which humans are exposed primarily through ingestion or contact [40]. Chronic low-dose exposure to these compounds has been associated with several cardiovascular risk factors and adverse cardiometabolic outcomes [41,42,43]. Bioinformatic analyses identified inflammatory signaling among the major pathways potentially involved in pesticide mixture-associated atherogenesis, including TNF and phosphatidylinositol 3-kinase (PI3K)/protein kinase B (Akt) signaling, while *IL6*, *TNF*, and *PTGS2* (encoding cyclooxygenase-2, implicated in prostaglandin synthesis that modulates vascular inflammation and contributes to atherosclerosis and thrombosis [44]) emerged among candidate hub genes potentially linking pesticide exposure to ASCVD [14].

Phthalates are ubiquitous plasticizers widely used in a variety of consumer products and represent a major source of human exposure to EDCs, mainly through contaminated food [45]. Growing epidemiological and experimental evidence suggests a role for phthalates in CVD development, including mechanisms relevant to atherosclerosis [16]. Using an integrated epidemiological and computational approach, Gong et al. identified candidate inflammation-related pathways, including Janus kinase/signal transducer and activator of transcription (JAK–STAT) signaling, as potential mechanisms underlying phthalate-associated cardiovascular toxicity [46]. Given its central role in regulating inflammatory mediator expression, JAK–STAT signaling further supports the contribution of inflammation to the cardiovascular effects of phthalate mixtures [47].

In rats fed a high-fructose diet, co-exposure to concentrated ambient particulate matter (CAPs) and ozone (O_3_), two well established risk factors for CVD, promoted macrophage infiltration in epicardial adipose tissue, a visceral fat depot surrounding the coronary arteries, increased the expression of pro-inflammatory mediators including *Tnfα*, macrophage/monocyte chemoattractant protein-1 (*Mcp1*), and leptin, and reduced the expression of the anti-inflammatory genes *Il10* and adiponectin [48,49,50]. However, co-exposure did not consistently induce stronger inflammatory responses than single-pollutant exposures [48]. Phipps et al. further observed that exposure to mixed vehicle emissions promoted a pro-inflammatory signaling profile in adipose tissue, characterized by increased expression of the inflammatory mediators *Il6* and *Mcp1*, as well as increased leptin expression in high-fat diet-fed mice [51]. Consistent with these findings, a randomized crossover study in healthy elderly subjects showed that combined exposure to house dust and O_3_ increased IL-8 mRNA expression in peripheral blood mononuclear cells, consistent with vascular inflammatory processes involved in atherosclerosis [52,53]. Notably, a comparable pro-inflammatory response was not observed following exposure to house dust or O_3_ alone but only under co-exposure conditions [52]. In contrast, Aragon et al. reported that serum obtained from mice exposed to road dust induced endothelial expression of several inflammatory mediators, including IL-6, MCP-1, C-C motif chemokine ligand 5 (CCL5) and C-X-C motif chemokine ligand 1 (CXCL1), whereas co-exposure to road dust and O_3_ was associated mainly with increased MCP-1 expression [54].

Shan et al. investigated the atherogenic effects of co-exposure to 2,3,7,8-tetrachlorodibenzo-p-dioxin (TCDD) and polychlorinated biphenyls (PCBs), two POPs that frequently co-occur in the environment [55]. Due to their persistence, bioaccumulation, and widespread human exposure, mainly through contaminated food, both contaminants continue to pose a public health concern and have been associated with CVD and atherosclerosis [56,57,58]. Co-exposure to TCDD and Aroclor 1254, a representative mixture of more than 60 PCB congeners, exacerbated atherosclerotic lesion development in apolipoprotein E (ApoE)-deficient mice, a well-established model of experimental dyslipidemia and atherosclerosis, as evidenced by more severe lesions in both the aortic valve and aortic arch compared with individual exposures [55,59]. This aggravated vascular injury was accompanied by markedly increased circulating and hepatic levels of MCP-1, enhanced macrophage recruitment, accumulation of the platelet-derived chemokine platelet factor 4 within atherosclerotic lesions, and upregulation of the innate immune receptor retinoic acid-inducible gene-I, which has been shown to induce IL-8 production in atherosclerosis [55,60].

Further support for the involvement of inflammatory pathways was provided by a study conducted in low-density lipoprotein (LDL) receptor-deficient mice, a hyperlipidemic model characterized by elevated LDL cholesterol levels and susceptibility to aortic lesion development, fed an atherogenic diet and exposed to a PFAS mixture [61]. PFAS are a class of persistent and bioaccumulative environmental pollutants widely used in industrial applications and consistently associated with cardiovascular toxicity [17,62]. Although canonical inflammatory cytokines were not significantly altered, PFAS exposure induced extensive transcriptional remodeling in aortic macrophages, including upregulation of the pro-inflammatory chemokines *Cxcl2* and *Cxcl17*, consistent with enhanced immune cell recruitment and early inflammatory activation [61]. Complementary evidence from network toxicology analyses further suggested a role for inflammatory mechanisms in PFAS-associated atherosclerosis, identifying IL-10 and caspase-1 among candidate immune- and inflammation-related targets potentially linking PFAS mixtures to vascular inflammation [63]. Conversely, co-exposure to perfluorooctane sulfonic acid (PFOS), a POP listed under the United Nations Stockholm Convention because of its environmental persistence and adverse health effects, and PCB126, a dioxin-like coplanar congener, did not affect *Tnfα* gene expression in a mouse model, indicating no detectable activation of inflammatory pathways despite evidence of vascular toxicity associated with the mixture (see following sections) [64,65].

A cross-sectional study investigating co-exposure to 56 serum trace elements provided additional evidence for a possible link between metal mixtures, inflammation, and atherosclerosis-related processes [66]. Mixture analyses showed positive associations with both acute MI prevalence and severity, while mediation analyses identified high-sensitivity C-reactive protein as a significant mediator, particularly for disease severity, where inflammation accounted for nearly half of the overall mixture effect [66]. Similarly, NF-κB pathway cytokines mediated part of the association between exposure to a redox-related metal mixture and both obstructive coronary artery disease and adverse cardiovascular outcomes, further highlighting the contribution of inflammation to mixture-related cardiovascular toxicity [67]. At the tissue level, Ma et al. reported that As and fluoride (F), elements for which drinking water represents a major source of exposure and which have each been individually associated with CVD, increased the expression of MCP-1, IL-8, and IL-6 in rabbit aorta, supporting enhanced leukocyte recruitment into early atherosclerotic lesions and vascular inflammatory responses [68,69,70]. Notably, the magnitude of these effects was lower than that observed following exposure to either contaminant alone, indicating that the interaction between the two toxicants may not be additive [68]. In contrast, exposure to As and Cd, the latter being an established environmental risk factor for atherosclerosis and CVD primarily acquired through inhalation and the consumption of contaminated food and water, did not induce a significant inflammatory response in murine macrophages, whether administered individually or in combination [71,72]. The absence of changes in TNF-α, IL-1β, and IL-6 levels suggests that co-exposure did not potentiate inflammatory signaling beyond the effects of the individual metals [71] (Table 1).

### 3.2. Oxidative Stress

Oxidative stress, defined as an imbalance favoring increased generation of reactive oxygen species (ROS) and/or impaired antioxidant defense systems, is a key mechanism in the initiation and progression of atherosclerosis [73]. Excessive ROS production disrupts vascular redox homeostasis, leading to endothelial dysfunction, activation of pro-inflammatory signaling pathways, and lipid oxidation [73]. Furthermore, ROS promote the formation of oxidized LDL, which enhances monocyte recruitment, foam cell formation, vascular smooth muscle cell remodeling, and plaque progression [73]. In line with these mechanisms, increasing evidence indicates that environmental mixtures can disrupt redox homeostasis and activate oxidative stress pathways involved in vascular injury and atherosclerotic disease. Supporting the role of oxidative stress in mixture-induced vascular toxicity, He et al. reported that exposure of human umbilical vein endothelial cells (HUVECs) to a mixture of 16 priority-controlled PAHs increased ROS generation and oxidative DNA damage, as reflected by elevated 8-hydroxy-2′-deoxyguanosine (8-OHdG) levels, a marker of oxidative DNA damage [30]. Concomitantly, alterations in antioxidant defense systems were observed, including reduced superoxide dismutase activity and activation of the nuclear factor erythroid 2-related factor 2 (Nrf2), the master regulator of antioxidant defense and its downstream effector heme oxygenase-1, indicating disruption of cellular redox homeostasis [30,74].

These findings are consistent with those reported by Zhang et al., who identified oxidative damage as a potential mediator of the association between co-exposure to a mixture of bisphenols, parabens, triclosan (TCS), and triclocarban—high-volume additives used respectively in the production of polycarbonate and epoxy resins, as preservatives, and as antimicrobial agents—and IHD risk [75]. Despite their short half-lives, these compounds have emerged as EDCs of growing concern and have been implicated in cardiovascular toxicity [76,77,78,79]. Mixture analyses showed that combined exposure was associated with increased IHD risk and higher urinary 8-OHdG levels [75]. Mediation analyses identified oxidative DNA damage, reflected by 8-OHdG, as a potential mediator of the associations between several EDCs (bisphenol A—BPA, bisphenol F, bisphenol AP, and TCS) and IHD risk [75].

Co-exposure to CAPs and O_3_ in rats fed a high-fructose diet increased inducible nitric oxide synthase (iNOS) expression and reduced mitochondrial area in the epicardial adipose tissue, indicating enhanced oxidative stress and mitochondrial impairment [48]. The observed iNOS upregulation may have further contributed to oxidative and nitrosative stress through increased peroxynitrite formation and reduced nitric oxide bioavailability, thereby promoting vascular injury [80]. However, these effects were not consistently greater than those induced by CAPs or O_3_ alone [48]. Similarly, exposure to mixed diesel and gasoline vehicle emissions increased ROS generation in the aortic vasculature of mice [51]. ROS levels were elevated in exposed low-fat diet-fed mice compared with controls and were further increased in high-fat diet-fed animals, although no significant interaction between exposure and diet was detected [51].

In agreement with the existing literature identifying particulate-bound metals as important drivers of PM_2.5_-associated cardiovascular effects, Zhang et al. showed that exposure of cardiomyocytes to PM_2.5_-associated metal mixtures induced a concentration-dependent increase in ROS production [81,82]. Oxidative stress was accompanied by increased expression of iNOS and was markedly attenuated by pretreatment with the antioxidant N-acetyl-L-cysteine, a well-established antioxidant that replenishes intracellular glutathione stores, supporting a key role for oxidative mechanisms in PM_2.5_ metal-induced cardiotoxicity [81,83]. Evidence of oxidative stress induction following multipollutant co-exposure was also reported in a randomized crossover study conducted in healthy elderly subjects [52]. Combined exposure to house dust and O_3_ increased the ROS production capacity of circulating monocytes and granulocytes and upregulated the expression of 8-oxoguanine DNA glycosylase, a key enzyme involved in the base excision repair of 8-oxoguanine, thereby maintaining genomic stability [52,84]. Additionally, Zhang et al. reported a positive association between co-exposure to the dinitroaniline herbicides trifluralin and pendimethalin and 10-year ASCVD risk [85]. Although no overall association was observed between the herbicide mixture and mitochondrial DNA copy number (mtDNAcn), a biomarker of mitochondrial dysfunction and oxidative DNA damage, lower mtDNAcn was associated with higher trifluralin exposure and partially mediated the association between trifluralin and ASCVD risk, suggesting a potential role for mitochondrial damage in herbicide-related cardiovascular effects [85].

These findings are further supported by a large NHANES study, which reported that the combined effect of volatile organic compounds (VOCs) and heavy metals, two contaminant classes independently linked to CVD through oxidative, inflammatory, and metabolic mechanisms, was associated with a lower Oxidative Balance Score (OBS), an indirect indicator of systemic redox status reflecting the balance between pro-oxidant and antioxidant influences [86,87,88]. Lower OBS was, in turn, associated with greater odds of CVD [86]. Mediation analyses further identified the triglyceride–glucose index (TyG), atherogenic index of plasma, Castelli risk index II, and non-high-density lipoprotein (HDL) cholesterol as partial mediators of the OBS–CVD association [86].

In mice exposed to PCB126, PFOS, or their combination, hepatic expression of *Nrf2* was increased in all exposure groups, whereas NAD(P)H:quinone oxidoreductase 1 (*Nqo1*), encoding a cytoprotective enzyme involved in cellular antioxidant defense, was significantly upregulated only in the mixture-exposed group, with evidence of additive synergism between PCB126 and PFOS [64,89]. Despite these changes, hepatic total antioxidant capacity remained unchanged, possibly reflecting compensation by Nrf2- and Nqo1-mediated antioxidant responses [64].

At the cellular level, As and F exposure increased ROS generation, lipid peroxidation, NADPH oxidase (NOX) activity, and expression of p22phox, an essential component required for NOX function, in HUVECs [90]. However, the oxidative response to co-exposure was attenuated compared with F alone, indicating an antagonistic interaction between the two toxicants [90,91]. In contrast, low-dose co-exposure to As and Cd did not significantly increase macrophage ROS generation despite evidence of cell-type-specific interactions between the two contaminants [72]. Further evidence comes from Fang et al., who identified a redox-related metal mixture (nickel, zirconium, titanium, strontium, and vanadium) associated with increased oxidation–reduction potential, higher levels of protein carbonyls, malondialdehyde, and 8-OHdG, obstructive coronary artery disease, and adverse post-percutaneous coronary intervention outcomes in patients with type 2 diabetes [67]. NF-κB-related cytokines partially mediated these associations, linking oxidative stress to downstream inflammatory processes [67]. A recent cross-sectional study found that a urinary metal(loid) mixture composed of As, copper, manganese, and vanadium was associated with increased oxidative stress, as indicated by higher levels of advanced oxidation protein products, methylglyoxal, myeloperoxidase, and arginase, biomarkers that have been implicated in oxidative stress, endothelial dysfunction, atherosclerosis, and CVD [92,93,94,95,96]. Although the metal(loid) mixture was not directly linked to cardiovascular risk, an increased oxidative stress burden, assessed using a composite oxidative stress index, was positively associated with cardiovascular susceptibility [92] (Table 2).

### 3.3. Lipid Dysregulation

Lipid dysregulation is a fundamental driver of atherosclerosis. Among the established risk factors, dyslipidemia—particularly, elevated LDL cholesterol and reduced HDL cholesterol—plays a central role in atherosclerosis progression [97]. Furthermore, specific lipoprotein subfractions, including small dense LDL particles and oxidized LDL, have emerged as important determinants of ASCVD risk due to their increased atherogenic potential [61]. Beyond circulating lipid abnormalities, alterations in adipose tissue function and cellular lipid metabolism may also contribute to atherogenesis by promoting foam cell formation, vascular inflammation, and metabolic dysfunction [98].

Consistent with this hypothesis, recent studies have reported alterations in both adipose tissue metabolic regulation and circulating lipoprotein profiles following exposure to environmental contaminant mixtures and co-exposures. Sun et al. reported that short-term inhalational exposure to CAPs and O_3_ altered metabolic programming in epicardial adipose tissue of high-fructose-fed rats, suggesting disruption of adipose tissue metabolic homeostasis [48]. Further evidence of adipose tissue-related lipid dysregulation was provided by an in vivo study reporting increased epididymal fat pad weight and adipocyte hypertrophy in mice exposed to mixed vehicle emissions [51]. These changes, together with reduced GLUT4 and insulin receptor expression, particularly in high-fat diet-fed animals, were indicative of impaired adipose tissue metabolic function and insulin signaling [51]. In humans, a recent panel study in healthy young adults showed that exposure to a PM_2.5_-bound metal mixture was associated with extensive metabolomic alterations, particularly involving sphingolipid and fatty acid pathways, with antimony identified as the major contributor [99]. Several ceramide- and sphingolipid-related metabolites were also associated with blood pressure and endothelial dysfunction markers, suggesting a link between metal mixture-induced metabolic alterations and early cardiovascular injury [99].

In LDL receptor-deficient mice fed an atherogenic diet, PFAS mixture exposure increased circulating cholesterol-rich lipoproteins and induced lipid metabolism-related transcriptional changes in aortic macrophages, consistent with a transition toward a foam cell-like, pro-atherogenic phenotype [63]. Complementary computational analysis identified peroxisome proliferator-activated receptor γ (PPARγ) as a putative core molecular target of PFAS-associated atherosclerosis [63]. Given the central role of PPARγ signaling in lipid metabolism, adipogenesis, and macrophage lipid handling, dysregulation of this pathway may contribute to foam cell formation and atherogenesis [100]. In line with these observations, co-exposure to PFOS and PCB126 induced marked hepatic lipid dysregulation in a mouse model, characterized by increased lipid accumulation, elevated cholesterol ester levels, and accumulation of oxidized phospholipids (OxPLs) [64]. Importantly, co-exposure was associated with significant interactions affecting several OxPLs [64]. These alterations were accompanied by increased levels of atherogenic biomarkers, further linking pollutant-induced lipid remodeling to cardiovascular risk (see Section 3.4) [64]. A similar involvement of lipid metabolic pathways emerged from a nested case–control study of patients with type 2 diabetes undergoing percutaneous coronary intervention, which identified 110 PFAS-associated lipid species, including 55 significant mediators of PFAS-associated cardiovascular risk [101]. Notably, glycerophospholipids represented the largest proportion of mediating lipids, with some species accounting for more than 40% of the overall PFAS effect [101].

Similarly, Zhang et al. reported that co-exposure to a mixture of bisphenols, parabens, TCS, and triclocarban was associated with reduced HDL concentrations [75]. Mediation analyses further suggested that HDL may mediate the association between EDC mixture exposure and IHD risk, supporting the involvement of disrupted lipoprotein metabolism in EDC-associated cardiovascular disease [75]. Additional epidemiological evidence showed that exposure to heavy metal mixtures was associated with higher TyG, a surrogate marker of insulin resistance [102]. Zn was identified as the main contributor to these associations, whereas white blood cells, together with monocytes and lymphocytes, acted as key mediators, suggesting that inflammation may contribute to heavy metal-related metabolic dysfunction.

A case–control study investigating combined exposure to PAHs, organophosphate flame retardants (OFR), and phthalate acid esters (PAE) revealed a discrepancy between mixture- and compound-level findings [103]. While mixture-based analyses showed positive associations with IHD risk, glucose–lipid metabolism did not significantly mediate the overall mixture effect [103]. Conversely, significant mediation through metabolic pathways was observed for selected individual chemicals [103] (Table 3).

### 3.4. Endothelial Dysfunction

Endothelial cell injury and dysfunction represent an early and critical step in the development of atherosclerosis [104]. Dysfunctional endothelial cells exhibit enhanced expression of adhesion molecules and cytokines, promoting the recruitment of monocytes and other immune cells into the vascular wall [104]. These processes, in turn, contribute to lipid accumulation, foam cell formation, and subsequent plaque development [104]. Consistent with this paradigm, environmental mixtures and multipollutant co-exposures have emerged as important drivers of endothelial dysfunction and early vascular injury. Increased gene expression of adhesion molecules associated with vascular dysfunction, including vascular cell adhesion molecule 1 (VCAM-1) intercellular adhesion molecule 1 (ICAM-1,) and E-selectin, was observed in mice exposed to a PAH mixture compared with non-exposed animals, highlighting a potential role for PAH mixtures in promoting a pro-atherogenic endothelial phenotype [26]. Likewise, exposure to mixed vehicle emissions increased vascular expression of VCAM-1 and ICAM-1, with both adhesion molecules more highly expressed in high-fat diet-fed mice than in low-fat diet-fed and control animals, although a significant exposure–diet interaction was observed only for ICAM-1 expression [51]. Further evidence of endothelial dysfunction comes from a study in ApoE-deficient mice, in which co-exposure to CAPs and diesel exhaust gases (DEG)—a complex air pollution mixture containing carbonaceous particles, inorganic compounds, and PAHs—increased circulating VCAM-1 levels and enhanced vasomotor dysfunction [105]. However, no significant interaction between CAPs and DEG was observed with respect to atherosclerotic plaque progression [105].

Consistent with the hypothesis of endothelial injury, a randomized crossover study in healthy elderly subjects found that combined exposure to house dust and O_3_ significantly reduced circulating levels of late endothelial progenitor cells (EPCs), whereas neither exposure alone produced a significant effect [52]. Given the established role of EPCs in endothelial repair, re-endothelialization, and maintenance of vascular homeostasis [106], the observed reduction in late EPCs suggests that multipollutant exposure may impair endogenous vascular repair capacity and thereby contribute to cardiovascular risk. Additional evidence was provided by Aragon et al., who showed that serum from mice co-exposed to road dust and O_3_ increased endothelial ICAM-1 and VCAM-1 expression, whereas co-exposure to road dust and mixed vehicle emissions impaired acetylcholine-mediated vasorelaxation [54]. PM_2.5_-associated metal mixtures likewise induced dose-dependent ICAM-1 upregulation in cardiomyocytes, further supporting the activation of inflammatory pathways that may contribute to vascular injury and endothelial dysfunction across chemically diverse environmental mixtures [81].

In a study of participants with moderate-to-high CVD risk, urinary metabolites of acrolein and 1,3-butadiene were associated with impaired endothelial function, as indicated by lower reactive hyperemia index (RHI) values [107]. Specifically, higher levels of acrolein and 1,3-butadiene metabolites were associated with reduced RHI [107]. BKMR analyses further identified acrolein metabolite as the main contributor to the positive association between the VOC mixture and systolic blood pressure [107].

Furthermore, exposure of HUVECs to a mixture of 16 priority-controlled PAHs impaired endothelial migration and tube formation in a dose-dependent manner [30]. PAH-treated cells also exhibited elongated, fibroblast-like morphological changes and reduced chemotactic activity [30].

Evidence of endothelial injury was further provided by a study investigating co-exposure to BPA and its major substitutes BPF and bisphenol S [108]. Epidemiological evidence from NHANES linked urinary bisphenol mixtures to heart disease [108]. Complementary in vitro experiments in murine aortic endothelial cells showed that the bisphenol mixture reduced cell viability, increased Annexin V positivity, and activated caspase-3 and caspase-8, indicating enhanced apoptosis [108,109,110]. Compared with BPA alone, combined exposure shifted endothelial cell death towards apoptosis rather than RIP3/MLKL-dependent necroptosis [108,111].

In cultured HUVECs, a mixture of As and F increased the expression of VCAM-1, ICAM-1, and pentraxin 3, a marker implicated in vascular inflammation and atherosclerotic lesions, reduced NO production, and promoted endothelial apoptosis [90,112]. Notably, the induction of adhesion molecules was less pronounced following co-exposure than after F exposure alone, suggesting an attenuation of the endothelial response during co-exposure [90]. Non-additive interactions were previously observed in rabbit aorta, where chronic co-exposure to As and F resulted in lower VCAM-1 and P-selectin expression than that induced by either contaminant alone [68]. Consistent with these findings, no changes in VCAM-1 expression were observed in endothelial cells exposed to As and Cd, either individually or in combination [72]. Furthermore, in ApoE−/− mice, co-exposure to environmentally relevant concentrations of As and Cd did not significantly increase aortic plaque burden or markedly alter plaque composition, despite sex-specific effects observed following single-metal exposures [72].

Among 29 identified CVD-related targets, computational analyses of cardiovascular toxicity associated with a mixture of ten di-2-ethylhexyl phthalate metabolites identified *PIK3CA*, which encodes the catalytic subunit of phosphatidylinositol 3-kinase (PI3K), as a putative core molecular target [46]. Integrated epidemiological and bioinformatics analyses of pesticide mixtures likewise highlighted PI3K-Akt signaling as a candidate pathway potentially involved in ASCVD, with *AKT1* emerging as one of the major hub genes [14]. Given the central role of PI3K-Akt signaling in regulating endothelial cell survival, proliferation, and vascular homeostasis, these findings are consistent with the hypothesis that vascular dysfunction may contribute to pesticide mixture-associated cardiovascular toxicity [113].

Using data from NHANES 2005–2018 combined with network toxicology analyses, Pan et al. identified carbonic anhydrase 2, a protein involved in vascular calcification, as a potential molecular mediator of PFAS-associated vascular injury [63,114]. Consistently, co-exposure to PFOS and PCB126 increased hepatic expression of ICAM-1 and PAI-1, a marker of endothelial dysfunction and atherosclerosis, and elevated circulating PAI-1 levels in mice [64,115]. Notably, the two pollutants showed additive synergistic effects on PAI-1 expression, suggesting enhanced activation of pathways involved in endothelial dysfunction, thrombosis, and cardiovascular risk [64] (Table 4).

### 3.5. Epigenetic Modifications

Epigenetic modifications, heritable and reversible changes in gene regulation that occur without altering the underlying DNA sequence, play a key role in atherosclerosis, influencing disease development, progression, and complications beyond genetic predisposition [116,117]. Epigenetic mechanisms such as DNA methylation, histone modifications, and non-coding RNAs exert cell-specific effects [117]. Both global and locus-specific changes in DNA methylation and histone modifications regulate endothelial cell responses to atherogenic stimuli, while histone modifications also contribute to vascular smooth muscle cell plasticity during atherogenesis [117]. Accumulating evidence indicates that both abnormal hypermethylation and hypomethylation of DNA contribute to the pathogenesis of atherosclerosis [118]. In this context, Lin et al. investigated whether global DNA methylation contributes to the relationship between Pb/Cd co-exposure and subclinical atherosclerosis in adolescents and young adults [119]. Despite marked reductions in environmental exposure over recent decades, Pb remains an important cardiovascular toxicant [120,121]. Both metals were individually associated with increased carotid intima-media thickness (CIMT), a surrogate marker of atherosclerosis, and global DNA methylation, assessed as the ratio of 5-methyl-2′-deoxycytidine to 2′-deoxyguanosine; however, after mutual adjustment, only Pb remained significantly associated with both outcomes [119,122]. Structural equation modeling further suggested that DNA methylation partially mediated the relationship between Pb exposure and CIMT, supporting an epigenetic mechanism underlying Pb-related subclinical atherosclerosis [119].

Within a prospective cohort study, mixed exposure to PM_2.5_, PM_10_, NO_2_, and SO_2_—air pollutants consistently associated with cardiovascular morbidity and mortality—was significantly associated with incident CVD [123,124,125,126]. However, epigenetic Mendelian randomization analyses focused on CpG sites previously associated with individual pollutants rather than on mediation of the mixture effect [123]. PM_2.5_-related cg01065697 and NO_2_-related cg07091220 were associated with increased risks of MI, while cg01065697 was also linked to IHD [123]. These findings support a potential contribution of pollutant-related DNA methylation changes to cardiovascular disease susceptibility, although not specifically to the effects of the pollutant mixture.

Among non-coding RNAs, microRNAs (miRNA), a class of endogenous, evolutionarily conserved, single-stranded non-coding RNAs of 21–22 nucleotides in length, have emerged as key post-transcriptional modulators of gene expression, linking environmental exposures to vascular dysfunction and atherosclerosis-related processes [127]. Wahlang et al. were among the first to examine alterations in miRNA expression profiles associated with PCB exposure [128]. In HUVECs, the PCB mixture Aroclor 1260 significantly altered the miRNA expression profile, with 21 dysregulated miRNAs linked to vascular disease, including miR-21, miR-31, miR-126, miR-221, and miR-222, which have been implicated in inflammatory signaling, endothelial dysfunction, and vascular injury [128,129,130,131,132]. In ApoE−/− mice, TCDD and Aroclor 1254 exposure altered hepatic miRNA and mRNA expression profiles, identifying several dysregulated miRNAs predicted to be associated with cardiovascular development and atherosclerosis, including miR-26a-5p, miR-193a-3p, miR-30c-5p, miR-130a-3p, and miR-376a-3p [133]. These changes were accompanied by increased atherosclerotic lesion development, supporting a potential role for miRNA dysregulation in POP mixture-induced cardiovascular toxicity [133].

More recently, Zhang et al. investigated the individual and joint associations between urinary PAH metabolites and arterial stiffness-related circulating miRNAs in a longitudinal panel study [134]. Arterial stiffness, an indicator of early vascular aging, shares key pathogenic mechanisms with atherosclerosis, including endothelial dysfunction, extracellular matrix remodeling, and chronic low-grade inflammation [135]. BKMR analyses showed positive overall associations between PAH mixtures and circulating miR-222 and miR-146a, with 9-hydroxyfluorene emerging as the main contributor to the mixture effect [134].

In a longitudinal panel study of middle-aged adults, WQS regression revealed positive associations between a mixture of urinary phthalate metabolites and circulating levels of miR-146a, miR-125b, and miR-222, with mono-methyl phthalate and mono-n-butyl phthalate identified as the main contributors to the mixture effect [136]. Importantly, miR-146a was also inversely associated with ankle-brachial index (ABI), which was used as an indicator of vascular function, and mediation analyses suggested that miR-146a partly mediated the relationship between phthalate exposure and ABI, in line with clinical evidence showing that dysregulated miR-146a expression is associated with the severity of coronary lesions [136,137] (Table 5).

## 4. Evidence Integration and Mechanistic Insights

The overall evidence is strongest for oxidative stress, inflammatory activation, and endothelial dysfunction, for which convergent findings are available across human and experimental studies. Evidence for lipid dysregulation is increasingly supported but remains more heterogeneous, reflecting the diversity of metabolic endpoints investigated. By contrast, epigenetic mechanisms remain an emerging area of research and are supported by a relatively small number of studies, many of which rely on cross-sectional designs, surrogate markers, or limited functional validation. Therefore, the role of epigenetic alterations in mixture-associated atherosclerosis should currently be considered suggestive rather than definitively established.

Environmental mixtures may contribute to CVD, including ASCVD, through the activation of inflammatory pathways. PAH mixtures promote inflammatory responses at both the systemic and cellular levels [26,30,31]. These observations are biologically plausible and consistent with extensive experimental evidence showing that individual PAHs activate the aryl hydrocarbon receptor (AhR), a ligand-activated transcription factor involved in xenobiotic sensing, oxidative stress, and redox homeostasis [15,138]. AhR activation can interact with NF-κB signaling and promote pro-inflammatory responses, while AhR signaling can also enhance IL-17-related responses, thereby contributing to the amplification of vascular inflammation [139]. The findings reported for THM, pesticide and phthalate mixtures further support the concept that inflammation represents a common mechanistic pathway across chemically distinct environmental mixtures [14,34,46]. Indeed, the mediating role of inflammatory indices, together with the involvement of IL-17-, TNF- and JAK–STAT-related signaling pathways, suggests that different contaminant classes may converge on shared immune and inflammatory mechanisms that ultimately promote vascular injury and atherosclerosis. In addition, exposure to PFAS mixtures has been associated with transcriptional remodeling of aortic macrophages, including upregulation of chemokines, suggesting enhanced immune cell recruitment and early inflammatory activation within the vascular wall [61,63]. Interestingly, computational analyses identified caspase-1 as a potential molecular target of PFAS mixture-associated atherosclerosis, in line with experimental evidence that individual PFAS, including PFOS and perfluorooctanoic acid, can activate inflammasome-related pathways and promote caspase-1-mediated inflammatory responses [63,140]. Beyond vascular inflammation, environmental contaminants may also affect adipose tissue surrounding the coronary arteries, an increasingly recognized contributor to CVD and coronary atherosclerosis, and a metabolically active visceral fat depot [141]. Evidence from air pollution co-exposure and mixed vehicle emission studies suggests that pollutant-induced adipose inflammation may contribute to atherosclerosis through the release of pro-inflammatory mediators and amplification of local and systemic inflammatory responses [48,51]. Notably, systemic inflammation also emerged as a potential mechanism underlying the cardiovascular effects of trace element mixtures [66]. Together with previous reports associating heavy metal exposure with inflammation- and oxidative stress-related pathways, these findings support a role for chronic inflammatory activation in the transition from atherosclerotic disease to acute MI as well as in adverse cardiovascular outcomes. [66,67,142,143,144].

Despite substantial differences in chemical composition, the available evidence suggests convergence toward redox imbalance, which can amplify inflammatory signaling, impair endothelial integrity, and disrupt vascular homeostasis. This concept is supported by experimental findings showing increased ROS generation and oxidative damage following exposure to PAH and inorganic contaminant mixtures. In addition to PAH-induced oxidative DNA damage and alterations in antioxidant defenses, As-F co-exposure increased lipid peroxidation and NADPH oxidase activation, whereas other metal(loid)- and air pollution-related mixtures were associated with enhanced oxidative stress responses, including increased iNOS expression and other biomarkers linked to endothelial dysfunction and CVD, collectively supporting the contribution of redox imbalance to mixture-induced vascular injury across chemically distinct contaminant classes [30,48,81,90,92]. Of particular interest, EDC mixtures not only induce oxidative DNA damage but also show evidence that oxidative stress may lie on the pathway linking exposure to IHD risk [75]. The observed non-monotonic dose–response relationship further underscores the unique toxicological behavior of EDC mixtures, suggesting that even low-dose exposures may perturb redox homeostasis and contribute to cardiovascular toxicity [75,145]. Consistent evidence of redox imbalance has also been reported in humans following combined exposure to house dust and O_3_, supporting the relevance of oxidative stress pathways across experimental and clinical settings [52]. In addition, evidence showing reduced OBS, an integrated measure of redox homeostasis that reflects the combined influence of multiple pro-oxidant and antioxidant determinants, following co-exposure to heavy metals and VOCs, together with the association between lower OBS and CVD, further reinforces the contribution of systemic oxidative imbalance to mixture-related cardiovascular toxicity [86]. The relatively limited contribution of atherogenic indices to this association also suggests that OBS may capture additional biological processes involved in CVD beyond lipid-related pathways. In parallel, oxidation–reduction potential, a novel marker of global redox status, identified a redox-related metal mixture associated with increased oxidative damage, higher odds of obstructive coronary artery disease, and poorer cardiovascular outcomes [67]. Collectively, the recurring observation of oxidative stress across in vitro, animal, and human studies, together with evidence of oxidative DNA damage, mitochondrial dysfunction, and systemic redox imbalance, supports the hypothesis that redox dysregulation is a central contributor to atherogenesis and a point of convergence for multiple mechanisms involved in mixture-related cardiovascular toxicity.

Lipid dysregulation represents an additional mechanism linking environmental mixtures to atherosclerosis. It should be noted that the available evidence includes both direct measures of lipid dysregulation, such as circulating lipoproteins, cholesterol fractions, oxidized lipids, and lipidomic profiles, and indirect or surrogate indicators, including TyG, adipose tissue remodeling, metabolomic signatures, and pathway-based analyses. These endpoints capture different aspects of metabolic dysfunction and should not be considered fully interchangeable when interpreting the evidence. Findings from PFAS mixture studies are consistent with the growing body of epidemiological evidence associating individual PFAS compounds with dyslipidemia, including alterations in cholesterol concentrations, lipoprotein subfractions, and apolipoprotein profiles [146,147,148]. Beyond these systemic effects, PFAS exposure has been shown to modulate genes involved in lipid metabolism, fatty acid transport, synthesis, and storage, and to induce broad lipidomic perturbations involving glycerophospholipids, glycerolipids, sphingolipids, acylcarnitines, and OxPLs, indicating that disruption of lipid homeostasis may represent a central component of PFAS-related cardiovascular toxicity [61,64,101]. The involvement of PPAR signaling provides a biologically plausible explanation for the lipid alterations observed following PFAS mixture exposure, given the central role of PPAR pathways in lipid metabolism and adipogenesis, and is consistent with previous evidence that individual PFAS compounds disrupt lipid homeostasis through PPAR-mediated mechanisms [63,149]. In addition, co-exposure to PFOS and PCB126 produced additive or multiplicative interactions for several OxPL species, suggesting enhanced oxidative lipid remodeling that may contribute to cardiovascular toxicity through liver–vascular crosstalk mechanisms [64,150,151]. In parallel, alterations in adipose metabolic genes following air pollution co-exposure, together with adipocyte hypertrophy, impaired insulin signaling, and increased expression of 5enin–angiotensin system (RAS) components following mixed vehicle emission exposure, support the emerging role of adipose tissue dysfunction in cardiometabolic disease and atherosclerosis [48,51,98,152]. Given the established role of adipose RAS in metabolic dysfunction, the observed increases in angiotensinogen and angiotensin II receptor type 1 (AT1) expression further implicate this pathway as a potential mechanism linking traffic-related pollutant mixtures to adipose tissue remodeling and cardiometabolic risk [51,153]. Comparable metabolic alterations were also observed following PM_2.5_-bound metal mixtures, which induced sphingolipid remodeling, indicating that metabolic remodeling may represent a shared mechanism linking chemically distinct environmental mixtures [100,154]. Beyond alterations in adipose tissue homeostasis, evidence from EDC mixtures suggests that their atherogenic effects may arise from multiple interconnected mechanisms, including lipid dysregulation, oxidative stress, and perturbations of estrogenic signaling, underscoring the multifactorial nature of their potential contribution to atherogenesis [75]. Collectively, these observations suggest that environmental mixtures might affect lipid metabolism at multiple levels, ranging from adipose tissue homeostasis to circulating lipoproteins and macrophage lipid handling, thereby promoting lipid accumulation, foam cell formation, and a pro-atherogenic metabolic milieu.

In the context of endothelial dysfunction, mixtures of PAHs, VOCs, traffic-related air pollution, and inorganic contaminant mixtures impair vascular repair mechanisms, compromise endothelial function, and induce endothelial injury, thereby favoring the development of a pro-atherogenic endothelial phenotype [26,30,51,90,107]. Beyond endothelial activation, environmental mixtures may directly promote endothelial injury through the induction of regulated cell death pathways. Notably, a bisphenol mixture induced endothelial apoptosis, whereas BPA alone had previously been associated with necroptotic signaling, highlighting how co-exposure may modify the underlying mechanisms of vascular toxicity [108,155]. Furthermore, the endothelial alterations induced by these mixtures occurred in parallel with oxidative stress and inflammatory activation, supporting the existence of interconnected mechanisms contributing to endothelial injury and early atherogenic changes. Evidence from heavy metal-containing PM_2.5_ mixtures, air pollution co-exposure models and persistent organic pollutant mixtures further indicates that endothelial activation, impaired vascular function, and thrombosis-related endothelial injury represent overlapping mechanisms linking complex pollutant mixtures to atherosclerosis [30,64,81,105]. These effects may also involve activation of the vascular RAS, as mixed vehicle emission exposure increased aortic AT1 receptor expression, thereby implicating a pathway known to contribute to metabolic dysregulation, oxidative stress, endothelial activation, and vascular injury [51,156]. Computational analyses also identified the PI3K–Akt signaling pathway as a potential mediator of phthalate- and pesticide-associated cardiovascular toxicity [14,46]. These findings are consistent with experimental evidence showing that mono(2-ethylhexyl) phthalate can induce inflammatory and angiogenic alterations through PI3K/AKT signaling, supporting a potential role for endothelial dysfunction and altered vascular homeostasis as convergent mechanisms across chemically diverse environmental mixtures [157].

Epigenetic mechanisms may help explain the relationship between toxic metal exposure and atherosclerosis-related outcomes. Both Cd and Pb have individually been associated with alterations in global and locus-specific DNA methylation patterns in humans and experimental models, potentially through modulation of DNA methyltransferase activity [158,159]. Furthermore, the pro-oxidant properties of these metals may indirectly affect methylation profiles by reducing the activity of ten–eleven translocation enzymes, which oxidize 5-methylcytosine and promote locus-specific DNA demethylation [158,159]. However, despite the associations of both Pb and Cd with subclinical atherosclerosis, global DNA methylation emerged as a potential mediator only for Pb-related increases in CIMT, suggesting metal-specific epigenetic mechanisms [119]. Overall, POP mixtures, including dioxins, PCBs, PAHs, and phthalates, are associated with alterations in vascular-related miRNA expression profiles [128,133,134,136]. These observations support a role for epigenetic reprogramming as a potential link between complex environmental exposures and early atherosclerotic alterations, highlighting the contribution of mixture-driven mechanisms relevant to atherosclerosis. Notably, several of the dysregulated miRNAs identified across studies were associated with CVD–related networks and atherosclerosis signaling pathways, indicating a role for miRNA-mediated regulation in the cardiovascular effects of environmental contaminant mixtures. Such associations are biologically plausible, as diverse environmental contaminants converge on shared molecular pathways involving oxidative stress, inflammation, endocrine disruption, and epigenetic regulation, which collectively influence miRNA expression and cardiovascular homeostasis. Among these contaminants, PAHs are known to induce oxidative stress [160] and activate AhR signaling [161], both of which can modulate miRNA expression [162,163], while also interfering with endocrine pathways regulating vascular function [164,165]. Similarly, dioxins and dioxin-like PCBs promote oxidative stress [151,166], inflammation [151,167], and AhR-dependent signaling in vascular cells [168,169], further supporting a common mechanistic basis for miRNA dysregulation across different contaminant classes. Individual phthalate compounds have also been shown to induce oxidative stress, inflammation, and epigenetic alterations, including changes in miRNA expression, contributing to endothelial dysfunction and atherogenesis [16]. Collectively, the available evidence provides biological plausibility for the associations between phthalate mixtures and circulating vascular-related miRNAs. Taken together, current data suggest a possible biological framework in which environmental mixtures converge on shared molecular pathways—including oxidative stress, inflammation, and endocrine disruption—to contribute to miRNA-mediated epigenetic alterations, thereby linking cumulative exposures to vascular dysfunction, arterial stiffness, and early atherosclerotic changes. Importantly, miRNAs may represent both biomarkers of exposure and potential mediators of mixture-induced vascular injury, although further mechanistic validation is required.

Whether these pathways truly represent common biological hubs for environmental mixtures, however, remains uncertain. The apparent convergence on these pathways may reflect genuine biological vulnerability, but it may also arise from the way environmental health research has progressed. These pathways are among the best characterized mechanisms in cardiovascular biology and are supported by well-established experimental assays and biomarkers, making them natural targets for both mechanistic and observational investigations. In parallel, research has disproportionately focused on a relatively small group of well-characterized environmental chemicals, while thousands of other environmental chemical exposures remain largely unexplored. Together, these factors may create a reinforcing research cycle in which familiar chemicals are repeatedly examined through familiar mechanistic frameworks, further strengthening the perception that these pathways represent the dominant biological mechanisms while potentially overlooking alternative pathways affected by less-studied exposures.

## 5. Challenges and Knowledge Gaps

Despite the growing evidence linking environmental mixtures to atherosclerosis-related mechanisms, several limitations should be acknowledged. The reviewed evidence derives from conceptually distinct approaches, including controlled co-exposure experiments, multipollutant systems, and epidemiological studies applying statistical mixture methods such as WQS, BKMR, and quantile g-computation. Because these approaches address different aspects of mixture exposure and toxicity, their findings should be interpreted as complementary rather than directly comparable.

First, substantial heterogeneity exists across the investigated mixtures, which differ markedly in chemical composition, concentration ranges, exposure sources, and routes of exposure. Studies employed a wide range of mixture-analysis approaches, including BKMR, WQS regression, quantile-based g-computation, and other statistical frameworks, complicating direct comparisons across studies and potentially contributing to differences in reported findings. WQS regression does not directly capture complex non-linear relationships or interactions among pollutants, whereas BKMR can evaluate these complex exposure patterns, albeit with higher computational demands and potentially lower statistical power.

In addition, the available literature remains methodologically heterogeneous with respect to study design. While several studies were conducted in human populations, many relied on cross-sectional analyses, limiting causal inference, whereas prospective epidemiological investigations remain relatively scarce. Moreover, exposure assessment often relied on a single biological sample measurement, which may not adequately capture long-term cumulative exposure to persistent pollutants or account for the influence of exposure timing on cardiovascular outcomes. Comparisons across human studies are further complicated by the use of different biological matrices, including serum, plasma, whole blood, and urine, which may reflect distinct exposure windows and toxicokinetic properties. Furthermore, the populations investigated differed substantially with respect to age, geographic location, socioeconomic characteristics, environmental exposure patterns, baseline cardiovascular risk, individual genetic susceptibility, and healthcare systems, potentially limiting the comparability and generalizability of the findings across studies [170,171]. Additional heterogeneity arises from differences in covariate selection and confounder adjustment strategies, potentially contributing to inconsistencies in the reported associations.

Although experimental studies provide valuable mechanistic insights, several factors may limit their direct applicability to real-world human exposure scenarios. Some experimental studies employed exposure concentrations substantially exceeding human-relevant environmental levels. Although these models can facilitate the identification of molecular targets and biological pathways, their physiological relevance may be limited and caution is warranted when extrapolating such findings to human populations [30,55,64,90]. Furthermore, several in vivo studies employed genetically modified models, such as ApoE-deficient mice, and/or atherogenic diets to accelerate plaque development, experimental conditions that do not fully reproduce the natural history and complexity of human atherosclerosis [55,61,72]. Some studies also included limited numbers of animals per exposure group and focused exclusively on male animals, restricting statistical power and limiting the evaluation of sex-specific mechanisms and susceptibility [26,51]. This limitation is particularly relevant given the growing evidence that biological sex can influence cardiovascular susceptibility, inflammatory responses, oxidative stress pathways, endocrine signaling, and the toxicity of environmental contaminants. Consequently, potential sex-specific differences in the cardiovascular effects of environmental mixtures remain insufficiently characterized and should be considered a priority for future experimental and epidemiological research. In addition, important interspecies differences in the absorption, distribution, metabolism, bioaccumulation, and elimination of environmental contaminants may influence internal dose and biological responses, thereby limiting the direct translation of animal findings to human populations.

Finally, considerable variability exists in the biomarkers and cardiovascular endpoints assessed across studies. These outcomes span different levels of evidence, ranging from molecular and cellular biomarkers and surrogate markers of atherosclerosis to clinically manifest cardiovascular endpoints, and therefore should not be considered directly comparable when evaluating the strength of the available evidence. Oxidative stress, inflammation, endothelial dysfunction, lipid metabolism, and epigenetic alterations were frequently evaluated using different measures and methodological approaches, making quantitative synthesis and comparison of findings challenging. Moreover, some of the proposed molecular targets and pathways originate from network toxicology and bioinformatics studies. Although these approaches provide valuable insights into potential biological mechanisms, they are inherently hypothesis-generating and require experimental validation. Accordingly, such findings should be interpreted with appropriate caution.

An additional challenge emerging from the reviewed literature is that mechanistic effects identified for individual contaminants are not necessarily reproduced when exposures are evaluated as mixtures. This phenomenon is illustrated not only by the absence of glucose-lipid metabolism mediation for OFR/PAE/PAH mixtures despite compound-specific effects [103], but also by several co-exposure studies in which combined exposures attenuated, modified, or failed to reproduce biological responses observed for individual pollutants, including As-F, As-Cd, CAPs-O_3_, and other air-pollution mixtures [48,54,68,72,90]. Furthermore, mechanistic investigations often remain focused on individual mixture components even when health effects are demonstrated at the mixture level [123]. Conversely, other studies reported enhanced biological effects following co-exposure, indicating that interactions among mixture components may amplify toxicity beyond that induced by individual pollutants [52,55,64,108]. Several mechanisms may contribute to the weaker or antagonistic responses observed under co-exposure conditions. Mixture components may compete for common molecular targets, signaling pathways, or metabolic and detoxification systems, thereby reducing the biological activity of individual contaminants. In addition, toxicokinetic interactions affecting absorption, distribution, metabolism, or bioavailability, as well as adaptive or compensatory cellular responses, may attenuate the overall response to combined exposures. Collectively, these findings suggest that environmental mixtures can generate additive, antagonistic, or synergistic interactions, highlighting the limitations of extrapolating mixture-associated mechanisms from single-chemical studies.

A broader knowledge gap in environmental health sciences is that the vast majority of environmental exposures remain uncharacterized. Despite the large number of natural and anthropogenic chemicals to which we are exposed throughout life, only a small fraction has been systematically measured or evaluated for cardiovascular toxicity. As a result, current evidence is dominated by a relatively limited group of well-studied contaminants, potentially overlooking important contributors to atherosclerosis. Moreover, the interpretation of environmental mixture analyses depends fundamentally on which exposures are included in the mixture. Because the composition of measured mixtures varies across studies, populations, and analytical platforms, mixture effects may reflect only a subset of the relevant chemical environment, complicating comparisons across studies and potentially obscuring the contributions of unmeasured co-exposures. Continued development and application of exposomic approaches will be critical for identifying previously unrecognized exposures, more comprehensively characterizing real-world mixtures, and advancing our understanding of environmental influences on cardiovascular disease. Despite growing interest in the cardiovascular effects of micro- and nanoplastics, no eligible studies specifically investigating these contaminants within environmental mixtures or multipollutant co-exposure scenarios were identified in the present review. This represents an important gap in the current literature and warrants further investigation.

From a public health perspective, these findings also highlight the limitations of risk assessment frameworks based predominantly on single-contaminant evaluations. Because real-world exposures occur as complex mixtures rather than isolated chemicals, approaches that consider combined exposures may provide a more realistic characterization of cardiovascular risk. This has potential implications for environmental monitoring, regulatory policies, and prevention strategies, supporting the progressive integration of mixture-oriented approaches into environmental health risk assessment.

**Figure 1:**
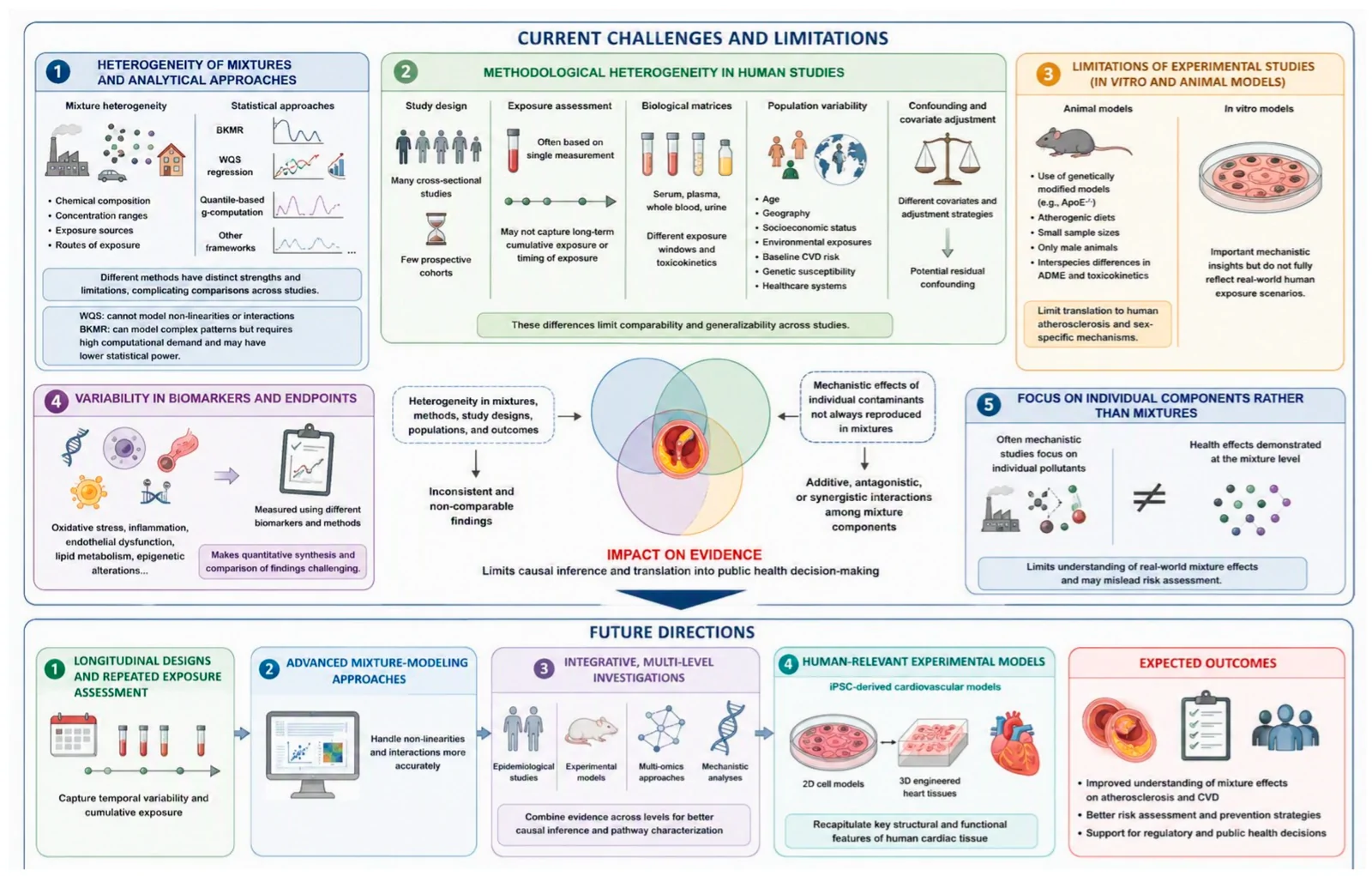
Graphical summary of the main challenges, knowledge gaps, and future research priorities in studies investigating the relationship between environmental mixtures and atherosclerosis. Abbreviations: BKMR: Bayesian kernel machine regression; WQS: weighted quantile sum. Image generated with the assistance of ChatGPT (OpenAI, GPT-5.5).

Future research should prioritize longitudinal designs, repeated exposure assessment, advanced mixture-modeling approaches, and integrative, multi-level investigations combining epidemiological studies, experimental models, multi-omics approaches, and mechanistic analyses to improve causal inference and better characterize the biological pathways linking long-term exposure to environmental mixtures with atherosclerosis and CVD. In parallel, the development and implementation of human-relevant experimental models will be essential to improve the translational value of mechanistic studies. In this context, induced pluripotent stem cell (iPSC)-derived cardiovascular models, including both two-dimensional and three-dimensional engineered heart tissues, may represent promising platforms for investigating the cardiovascular effects of environmental mixtures and multipollutant co-exposures, as they can recapitulate key structural and functional features of human cardiac tissue and retain donor-specific genetic backgrounds, thus enabling the study of interindividual variability in response to environmental exposures [172] (Figure 1).

## 6. Conclusions

Exposure to environmental mixtures has emerged as a potentially relevant contributor to atherosclerosis and cardiovascular toxicity. Despite substantial differences in chemical composition and exposure sources, the available evidence consistently indicates that complex mixtures promote vascular injury by inducing chronic inflammation, oxidative stress, lipid dysregulation, endothelial dysfunction, and epigenetic remodeling. Rather than acting through contaminant-specific pathways, diverse environmental mixtures appear to converge on a limited number of shared biological processes that collectively contribute to atherosclerotic disease initiation and progression. Nevertheless, this apparent convergence should be interpreted with caution, as it may partly reflect the predominance of well-studied contaminants, established mechanistic frameworks, and publication patterns favoring extensively investigated biological pathways.

Recent advances in mixture-oriented analytical approaches have substantially improved the ability to characterize the health effects of real-world environmental exposures. Nevertheless, important challenges remain, including methodological heterogeneity, limited longitudinal evidence, uncertainties regarding causal inference, and the need to clarify causal relationships and interactions among mixture components. Future large-scale prospective studies integrating exposomics, multi-omics technologies, and mechanistic experimental models will be essential to better characterize mixture-induced cardiovascular toxicity, identify reliable biomarkers of exposure and early vascular injury, and support the development of effective preventive strategies and evidence-based public health policies.

## Figures and Tables

**Table 1:** Environmental mixtures and co-exposures associated with inflammation-related mechanisms in atherosclerosis.

First Author and Year	Design	Model/Population	Exposure	Outcome	Key Findings	Relevance to Atherosclerosis
Rojas et al. 2022 [26]	Experimental in vivo study	Male BALB/c mice	PAH mixture (phenanthrene 55%, fluoranthene 25%, pyrene 20%); intranasal instillation (10 μL) at 10, 30, and 50 μg; 5 days/week for 5 weeks	Systemic inflammation	Increased serum IL-6 and IFN-γ. No significant changes in serum IL-10, IL-17A, TNF-α. No significant differences in aortic IL-6 or TNF-α gene expression.	PAH mixture–induced systemic pro-inflammatory response; early driver of atherogenesis.
He et al. 2022 [30]	Experimental in vitro study	Primary HUVECs	Mixture of 16 EPA priority PAHs; exposure concentrations derived from blood levels reported in the Chinese general population and tested at environmental (1×) and higher doses (10×–1000×); 48 h treatment duration	TNF-α expression and NF-κB signaling activation	Increased TNF-α expression across all PAH-treated groups. Increased NF-κB activation (p-p65 expression) in the highest exposure group (1000× PAHs).	PAH mixture–induced inflammatory activation; NF-κB/TNF-α signaling; pro-atherogenic vascular inflammation
Du et al. 2024 [31]	Cross-sectional study (NHANES 2003–2016)	9136 US adults; 10.5% with CVD	Urinary levels of 7 OH-PAHs metabolites (creatinine-adjusted)	Prevalent CVD (self-reported: IHD, angina, MI, stroke, heart failure)	Positive overall association between PAH mixture and CVD (BKMR); overall mixture effect increasing at exposure levels ≥ 55th percentile. 2-OHFlu as the main driver of the mixture effect (highest PIP) and strongest positive exposure–response relationship with CVD. NLR and SII mediation of 9.7% and 2.0% of the PAH mixture–CVD association, respectively.	PAH mixture–induced systemic inflammation contributing to CVD risk; supports a role for mixture-driven mechanisms in vascular injury and atherosclerosis development.
Zhao et al. 2025 [34]	Cross-sectional study (NHANES 2005–2012)	5400 US adults aged ≥45 years; 7.8% with CVD	Blood concentrations of THMs, TCM, TBM, BDCM, and DBCM	Prevalent CVD (self-reported: IHD, MI, angina, stroke)	Positive association between THM mixture and CVD risk in WQS analyses (OR = 1.16, 95%CI: 1.03–1.32) TCM as predominant contributor (58.0% mixture weight). Higher TCM concentrations (Q4 vs. Q1: OR = 1.42, 95%CI: 1.05–1.93) and TTHM concentrations (Q3 vs. Q1: OR = 1.57, 95%CI: 1.16–2.12) associated with increased CVD prevalence. NLR-mediated 7.12% of the TTHM–CVD association. Identification of 84 shared THM-, CVD-, and aging-related genes; enrichment of IL-17 signaling, fluid shear stress and atherosclerosis, AGE–RAGE signaling, and apoptosis-related pathways. Inflammation-related mechanisms and IL-17 signaling as potential links between THM exposure, vascular injury, atherosclerosis, and CVD.	THM mixture–induced systemic inflammation contributing to CVD risk. Involvement of IL-17–related inflammatory pathways and immune activation processes relevant to atherosclerosis progression.
Wu et al. 2025 [14]	Cross-sectional study integrating epidemiological analyses and toxicogenomic bioinformatics	2291 Chinese participants from rural areas	Mixed pesticide exposure (34 selected pesticides including neonicotinoids, organochlorine pesticides, organophosphorus pesticides, pyrethroids, and herbicides measured in blood and urine).	10-year ASCVD risk and inflammation-related molecular pathways	Total pesticide mixture exposure positively associated with 10-year ASCVD risk in QGC (OR = 3.223, 95%CI: 2.196–4.730) and WQS models (OR = 4.642, 95%CI: 3.070–7.020). Linear dose–response relationship between overall pesticide mixture exposure and high ASCVD risk. Identification of 112 pesticide-related atherosclerosis target genes; enrichment of TNF and PI3K-Akt signaling pathways; *IL6*, *TNF*, and *PTGS2* identified among the major hub genes linking pesticide exposure to ASCVD.	Pesticide mixture-associated inflammatory activation, including pathways implicated in atherogenesis and ASCVD development
Gong et al. 2025 [46]	Cross-sectional study (NHANES 2005–2018) integrated with network toxicology	12,127 US adults	Urinary concentrations of 10 DEHP metabolites	Prevalent CVD; network toxicology pathways	Positive association between phthalate mixture exposure and CVD (OR = 1.21, 95%CI: 1.07–1.37) (WQS). MEOHP identified as the main contributor to the mixture effect, followed by MECPP, MBzP, and MnBP. Identification of inflammation-related pathways, including JAK–STAT signaling.	Phthalate mixture-associated cardiovascular toxicity; involvement of inflammatory signaling pathways relevant to vascular inflammation and atherosclerosis.
Sun et al. 2013 [48]	Experimental in vivo study	High-fructose-fed male Sprague–Dawley rats	CAPs (356 ± 52 μg/m^3^) + O_3_ (0.485 ± 0.041 ppm), 8 h/day for 9 consecutive weekdays.	Inflammation in epicardial adipose tissue	Macrophage infiltration in epicardial adipose tissue. Increased *Tnfα*, *Mcp1*, and leptin expression. Reduced *Il10* and adiponectin expression. No consistent enhancement of inflammatory responses compared with single-pollutant exposures	CAPs and O_3_-induced epicardial adipose tissue inflammation, macrophage infiltration, and adipokine imbalance potentially contributing to vascular inflammation and atherogenesis.
Phipps et al. 2021 [51]	Experimental in vivo study	Male C57BL/6 mice fed LF or HF diet	MVE (diesel + gasoline exhaust; 100 μg PM/m^3^), whole-body inhalation, 6 h/day, 7 days/week, for 30 days	Vascular and adipose inflammation	Increased vascular MOMA-2 staining in MVE-exposed mice irrespective of diet. Increased adipose *Il6* and *Mcp1* expression in both LF- and HF-fed mice. Increased leptin expression restricted to HF-fed mice	Traffic-related air pollution mixture-induced vascular and adipose inflammation; enhanced immune cell recruitment; potential contribution to atherogenesis.
Jantzen et al. 2018 [52]	Randomized double-blind crossover study	23 healthy elderly subjects	House dust (275 μg/m^3^ PM_2.5_) + O_3_ (100 ppb), 5.5 h exposure	Systemic inflammatory signaling	House dust + O_3_ co-exposure, IL-8 mRNA expression increase of 59% (95%CI: 15–120%). O_3_ exposure alone, TNF mRNA expression reduction of 9% (95%CI: −16 to −0.8%) and MCP-1 mRNA expression reduction of 7% (95%CI: −12 to −2%). No significant inflammatory effects following house dust exposure alone.	House dust and O_3_ co-exposure–associated inflammatory activation, suggesting potential synergistic effects of combined air pollutant exposures.
Aragon et al. 2016 [54]	In vivo inhalation exposure with subsequent in vitro/ex vivo serum bioactivity assays	C57BL/6 mice	Acute inhalation exposure (6 h) to road dust (349 μg/m^3^ PM_2.5_), road dust + O_3_ (344 μg/m^3^ PM_2.5_ + 0.33 ppm O_3_), road dust + MVE; 342 μg/m^3^ PM_2.5_, MVE gases, MVE particulate matter (MVE-PM; 328 μg/m^3^ PM_2.5_), or wood smoke (380 μg/m^3^ PM_2.5_); serum collected 18–24 h post-exposure and incubated with murine cerebrovascular endothelial cells for 4 h.	Inflammatory potential of circulating factors	Road dust exposure: increased IL-6, CXCL1, MCP-1, and CCL5 expression. Road dust + O_3_ exposure: increased MCP-1 expression. No significant induction of inflammatory genes following MVE gases, MVE-PM, or road dust + MVE exposure. Attenuation of road dust-induced inflammatory responses following co-exposure to MVE gases, suggesting antagonistic interactions among mixture components	Air pollution mixture-associated circulating inflammatory mediators and vascular inflammatory activation
Shan et al. 2014 [55]	Experimental in vivo study	Male ApoE−/− mice	TCDD (15 μg/kg) + Aroclor 1254 (55 mg/kg; >60 PCB congeners) co-exposure via intraperitoneal injection four times over a 6-week period	Inflammation and innate immune activation	4.74-fold increase in circulating MCP-1 and 4.75-fold increase in hepatic MCP-1 following TCDD/Aroclor 1254 co-exposure. 5.74-fold increase in macrophage recruitment (CD68+). 3.7–5.9-fold increase in PF4 accumulation within atherosclerotic lesions. 1.83-fold increase in aortic *RIG-I* expression and 8.74-fold increase in hepatic RIG-I expression. Greater atherosclerotic lesion burden than individual exposures. No significant changes in plasma IL-6 or E-selectin levels.	TCDD/Aroclor 1254 mixture-associated inflammatory and immune activation relevant to atherogenesis.
Roth et al. 2026 [61]	Experimental in vivo study	Male Ldlr−/− mice fed an atherogenic diet	PFAS mixture (PFOA, PFOS, PFNA, PFHxS, GenX); 2 mg/L each in drinking water for 7 weeks	Inflammatory signaling in aortic macrophages	982 differentially expressed genes. Increased *Cxcl2* and *Cxcl17* expression. No significant changes in M1 marker (*Il1β* and *Tnfα*) and M2 marker (*Il4* and *Il10*) expression profile.	PFAS mixture-induced chemokine signaling and immune cell recruitment potentially contributing to vascular inflammation and atherogenesis.
Pan et al. 2025 [63]	Cross-sectional study (NHANES 2005–2018) integrated with network toxicology	1099 adolescents	Mixture of PFOS, PFOA, PFNA, and PFHxS assessed in serum	Inflammation	Positive associations of PFAS mixture exposure with AIP. Network toxicology identification of IL-10, TNF, and caspase-1 as core inflammation-related targets; shared PFAS–folate target caspase-1, suggesting inflammasome-mediated mechanisms relevant to atherosclerosis.	PFAS mixture–associated activation of inflammatory and inflammasome-related pathways potentially contributing to vascular inflammation and atherosclerosis.
Deng et al. 2020 [64]	Experimental in vivo study	Male C57BL/6 mice	Acute co-exposure to PCB126 (0.5 mg/kg) and PFOS (250 mg/kg) by intragastric gavage; evaluation after 48 h of exposure	Inflammatory signaling and vascular inflammation	Periportal inflammatory cell infiltration. No significant changes in hepatic *Tnfα* expression	Limited evidence of inflammation-related effects following PFOS–PCB126 co-exposure
Sun et al. 2025 [66]	Cross-sectional study	195 Chinese adults (94 AMI cases, 101 controls). AMI severity assessed by Gensini score.	Serum mixture of 56 trace elements; mixture analyses focused on Fe, Cu, Rb, Nb, Mo, Sb, and Ge.	AMI prevalence and severity	Positive associations of Cu and Rb with both AMI prevalence and severity. Inverse association of Fe with AMI prevalence. Positive association of Sb with AMI severity. Positive associations between trace element mixtures and both AMI prevalence (ERS OR = 2.72, 95%CI: 1.91–3.86) and severity (ERS OR = 1.94, 95%CI: 1.54–2.45). hsCRP-mediated effects accounting for 8.6% and 48.5% of the mixture effects on AMI prevalence and severity, respectively. hsCRP-mediated associations of low Fe (29.1%) and high Rb (15.6%) with AMI prevalence.	hsCRP-related inflammatory mechanisms potentially linking trace element co-exposure to atherosclerosis progression and AMI risk.
Fang et al. 2026 [67]	Cross-sectional study and prospective cohort study	3142 patients with T2DM, 1470 patients with obstructive CAD, 1212 patients undergoing PCI	Redox-related metal mixture identified through elementomic profiling, composed of Ni, Sr, Ti, V, and Zr	Inflammatory signaling	Higher TNF-α concentrations. Nominally higher IL-1β, IL-8, and MIP-1β concentrations. 22% increase in NF-κB pathway cytokines. NF-κB pathway cytokines mediating 9% of the association with obstructive CAD and 32% of the association with post-PCI MACCE.	Metal mixture-associated NF-κB-mediated inflammatory signaling linked to coronary atherosclerotic burden and adverse cardiovascular outcomes.
Ma et al. 2012 [68]	Experimental in vivo study	New Zealand White rabbits	As (13 mg/L As_2_O_3_), F (50 mg/L NaF), or combined As + F exposure via drinking water; 6-month exposure	Inflammation	Increased MCP-1 protein levels in As-, F-, and As+F-exposed rabbits. Increased IL-6 protein levels following As exposure. Upregulation of MCP-1, IL-8, and IL-6 mRNA expression. Lower induction of inflammatory mediators under co-exposure compared with single-contaminant exposure	As- and F mixture-induced inflammatory responses relevant to atherogenesis
Subramaniam et al. 2024 [71]	Combined in vitro and in vivo study	RAW264.7 macrophages, C166 endothelial cells, and ApoE−/− mice	Environmentally relevant low-dose As/Cd mixture (5–50 ppb As; 1.5–5 ppb Cd) in drinking water for 13 weeks in male and female ApoE−/− mice; parallel low-dose in vitro exposures	Inflammation	No significant inflammatory response observed in macrophages, with unchanged TNF-α, IL-1β, and IL-6 levels after exposure to As, Cd, or their combination. No increase in plaque macrophage content	No amplification of pro-atherogenic inflammatory signaling compared with individual-metal exposure.

Abbreviations: 2-OHFlu: 2-hydroxyfluorene; AIP: atherogenic index of plasma; AMI: acute myocardial infarction; ApoE: apolipoprotein E; As: arsenic; ASCVD: atherosclerotic cardiovascular disease; BDCM: bromodichloromethane; BKMR: Bayesian kernel machine regression; CAD: coronary artery disease; Cd: cadmium; CVD: cardiovascular disease; Cu: copper; Cxcl: chemokine (C-X-C motif) ligand; DBCM: dibromochloromethane; DEHP: di-2-ethylhexyl phthalate; EPA: Environmental Protection Agency; ERS: element risk score; F: fluoride; Fe: iron; Ge: germanium; GenX: ammonium perfluoro(2-methyl-3-oxahexanoate); HF: high-fat; HUVECs: human umbilical vein endothelial cells; hsCRP: high-sensitivity C-reactive protein; IFN-γ: interferon gamma; IHD: ischemic heart disease; IL: interleukin; MI: myocardial infarction; JAK-STAT: Janus kinase-signal transducer and activator of transcription; LF: low-fat; MACCE: major adverse cardiovascular and cerebrovascular events; MCP-1: macrophage/monocyte chemoattractant protein-1; MECPP: mono-2-ethyl-5-carboxypentyl phthalate; MIP-1β: macrophage inflammatory protein beta; MnBP: mono-n-butyl phthalate; Mo: molybdenum; MOMA-2: anti-monocyte + macrophage antibody; MVE: mixed vehicle emissions; Nb: niobium; NF-κB: nuclear factor kappa-light-chain-enhancer of activated B cells; NHANES: National Health and Nutrition Examination Survey; Ni: nickel; NLR: neutrophil-to-lymphocyte ratio; OH-PAHs: monohydroxylated polycyclic aromatic hydrocarbon; OR: odds ratio; PM: particulate matter; PAH: polycyclic aromatic hydrocarbon; PCB: polychlorinated biphenyl; PCI: percutaneous coronary intervention; PF4: platelet factor 4; PFAS: per- and polyfluoroalkyl substances; PFOA: perfluorooctanoic acid; PFHxS: perfluorohexanesulfonic acid; PFNA: perfluorononanoic acid; PFOS: perfluorooctane sulfonate; PI3K/Akt: phosphatidylinositol 3-kinase/protein kinase B; PIP: posterior inclusion probabilities; PTGS2: prostaglandin-endoperoxide synthase 2; QGC: quantile-based *g*-computation; Rb: rubidium; Sb: antimony; SII: systemic immune-inflammation index; Sr: strontium; TBM: bromoform; TCDD: 2,3,7,8-Tetrachlorodibenzo-p-dioxin; TCM: chloroform; THM: trihalomethane; Ti: titanium; TNF-α: tumor necrosis factor alpha; TTHM: total trihalomethanes; V: vanadium; WQS: weighted quantile sum; Zr: zirconium.

**Table 2:** Environmental mixtures and co-exposures associated with oxidative stress mechanisms in atherosclerosis.

First Author and Year	Design	Model/Population	Exposure	Outcome	Key Findings	Relevance to Atherosclerosis
He et al. 2022 [30]	Experimental in vitro study	Primary HUVECs	Mixture of 16 EPA priority PAHs; exposure concentrations derived from blood levels reported in the Chinese general population and tested at environmental (1×) and higher doses (10×–1000×); 48 h treatment duration	ROS production, oxidative DNA damage (8-OHdG), antioxidant defense (SOD), Nrf2/HO-1 signaling pathway.	Increased ROS levels following PAH mixture exposure, with peak levels observed after 24 h in most treatment groups. Increased 8-OHdG levels after 48 h exposure in all PAH-treated groups (1×–1000×). Reduced SOD activity after 48 h exposure in the 10×, 100×, and 1000× PAH groups. Increased expression of Nrf2 and HO-1 following PAH mixture exposure.	PAH mixture–induced oxidative stress; oxidative DNA damage and altered antioxidant defenses; redox imbalance relevant to atherogenesis.
Zhang et al. 2023 [75]	Case–control study	69 IHD cases, 146 controls	Co-exposure to bisphenols (BPA, BPF, BPS, BPAF, BPP, BPZ, BPAP), parabens (MeP, EtP, PrP, BuP), TCS, and triclocarban; urinary biomonitoring-based exposure assessment	Oxidative stress	Positive joint association between mixture exposure and IHD risk (OR = 1.52, 95%CI: 1.25–1.84). 2.22 ng/mL increase in urinary 8-OHdG per decile increase in mixture exposure. BuP, TCS, BPAP, and BPF as the main contributors to 8-OHdG elevation. 8-OHdG mediating 68.8% of the overall mixture–IHD association. Significant mediation of BPA-, BPF-, BPAP-, and TCS-associated IHD risk through 8-OHdG (5.73%, 9.10%, 19.8%, and 28.8%, respectively).	Endocrine-disrupting chemical mixture-induced oxidative stress relevant to atherosclerotic cardiovascular disease
Sun et al. 2013 [48]	Experimental in vivo study	High-fructose-fed male Sprague–Dawley rats	CAPs (356 ± 52 μg/m^3^) + O_3_ (0.485 ± 0.041 ppm), 8 h/day for 9 consecutive weekdays.	Oxidative stress in epicardial adipose tissue	Increased iNOS expression. Reduced mitochondrial area in epicardial adipose tissue. No consistent enhancement compared with single-pollutant exposures	CAPs and O_3_-induced oxidative stress and mitochondrial dysfunction in epicardial adipose tissue potentially contributing to vascular injury and atherogenesis.
Phipps et al. 2021 [51]	Experimental in vivo study	Male C57BL/6 mice fed low-fat or high-fat diet	MVE (diesel + gasoline exhaust; 100 μg PM/m^3^), whole-body inhalation, 6 h/day, 7 days/week, for 30 days	Vascular oxidative stress	Increased vascular ROS production (DHE staining) in MVE-exposed LF-fed mice compared with controls. Greater oxidative burden in HF-diet-fed mice exposed to MVE. No significant exposure-diet interaction.	Mixed vehicle emissions-induced vascular oxidative stress potentially contributing to endothelial injury and atherogenesis.
Zhang et al. 2016 [81]	Experimental in vitro study	H9C2 rat cardiomyocytes	Heavy metal-containing PM_2.5_ mixture (Cr, Ni, Cu, Cd, Pb, Zn, Mn, and Co) collected during different seasons in a coal-burning region of northern China; 0–10 μg/mL for 24 h	Oxidative stress	Dose-dependent ROS generation. iNOS increase 2.17-fold (spring PM_2.5_) and 4.64-fold (winter PM_2.5_). Attenuation of ROS-mediated responses following NAC pretreatment. ROS-dependent inflammatory and apoptotic signaling	PM_2.5_-associated metal mixture-induced oxidative stress relevant to cardiovascular toxicity and atherogenesis.
Jantzen et al. 2018 [52]	Randomized double-blind crossover study	23 healthy elderly subjects	House dust (275 μg/m^3^ PM_2.5_) + O_3_ (100 ppb), 5.5 h exposure	Systemic oxidative stress	Increased ROS production capacity in monocytes (+30%) and granulocytes (+25%). Increased *OGG1* mRNA expression (31%). Limited or absent responses following single-pollutant exposures.	Combined air pollutant exposure-induced systemic oxidative stress and oxidative DNA damage responses potentially contributing to endothelial injury and atherosclerosis.
Zhang et al. 2025 [85]	Community-based cohort study with cross-sectional and longitudinal analyses	Chinese community-dwelling adults from the Wuhan–Zhuhai cohort (*n* = 3399 cross-sectional participants; longitudinal panel of 158 participants with 363 observations).	Combined serum levels of dinitroaniline herbicides (trifluralin and pendimethalin)	10-year ASCVD risk; mtDNAcn	Trifluralin–pendimethalin co-exposure associated with increased 10-year ASCVD risk in BKMR and WQS analyses; WQS β = 0.092% (95%CI: 0.035–0.143); trifluralin as major contributor. Trifluralin exposure associated with increased ASCVD risk (β = 0.272%, 95%CI: 0.148–0.377) and lower mtDNAcn (β = −0.058, 95%CI: −0.104 to −0.012). Lower mtDNAcn associated with increased ASCVD risk (β = −0.298%, 95%CI: −0.528 to −0.068); mtDNAcn mediation of 3.8% of the trifluralin–ASCVD association. No overall mixture effect on mtDNAcn.	Herbicide-associated mitochondrial dysfunction and oxidative DNA damage as potential mechanisms contributing to ASCVD risk and atherogenesis
Scandino et al. 2026 [86]	Cross-sectional study (NHANES 2011–2020)	6516 adults aged ≥20 years	Combined exposure to 11 HMs (As, Ba, Cd, Co, Cs, Hg, Mn, Mo, Pb, Sn, W) and 11 VOC metabolites; urinary biomonitoring-based exposure assessment.	OBS and prevalent CVD	Combined HM+VOC exposure associated with lower OBS (higher oxidative stress). Strongest inverse associations observed for Cd and VOC metabolites including N-acetyl-S-(2-hydroxyethyl)-L-cysteine and N-acetyl-S-(2-carbamoyl-2-hydroxyethyl)-L-cysteine. Association observed across demographic subgroups and particularly pronounced among individuals aged 20–59 years, women, Mexican Americans, non-Hispanic Asians, and with the highest income. Higher OBS associated with lower odds of CVD (OR = 0.969, 95%CI: 0.959–0.979). Modest mediation of the OBS–CVD association by TyG, AIP, CRI-II, and non-HDL cholesterol (3.8–8.8%)	Environmental mixture-associated oxidative imbalance as a potential mechanism linking HM and VOC co-exposure to CVD, with possible interaction between oxidative stress and atherogenic/metabolic pathways
Deng et al. 2020 [64]	Experimental in vivo study	Male C57BL/6 mice	Acute co-exposure to PCB126 (0.5 mg/kg) and PFOS (250 mg/kg) by intragastric gavage; evaluation after 48 h of exposure	Redox balance	Upregulation of hepatic *Nrf2* in all exposure groups. Selective upregulation of hepatic *Nqo1* in mixture-exposed mice. Additive synergistic interaction between PCB126 and PFOS for *Nqo1* regulation. Unchanged hepatic TAC.	PCB126/PFOS mixture-associated oxidative stress and redox dysregulation contributing to vascular injury and atherosclerosis-related risk.
Ma et al. 2017 [90]	Experimental in vitro study	Primary HUVECs	As_2_O_3_, 5 μM and NaF, 1 mM, alone and in combination for 24 h	Oxidative stress	Increased ROS generation (1.6-, 2.0-, and 1.5-fold after As, F, and co-exposure, respectively). Increased lipid peroxidation (MDA). Increased NOX activity (109.1, 75.3, and 100.6 vs. 31.0 μM/min/mg in controls). Upregulation of *p22phox* expression (2.2-, 1.8-, and 1.4-fold, respectively). Less pronounced oxidative responses following co-exposure than fluoride alone, suggesting antagonistic interaction.	Association of As-F co-exposure with oxidative stress pathways implicated in atherosclerosis.
Subramaniam et al. 2024 [71]	Combined in vitro and in vivo study	RAW264.7 macrophages, C166 endothelial cells, and ApoE−/− mice	Environmentally relevant low-dose As/Cd mixture (5–50 ppb As; 1.5–5 ppb Cd) in drinking water for 13 weeks in male and female ApoE−/− mice; parallel low-dose in vitro exposures	Oxidative stress	No significant increase in ROS production following As, Cd, or combined exposure. Synergistic interactions in macrophages and antagonistic interactions in endothelial cells. Absence of enhanced oxidative responses following co-exposure compared with individual-metal exposure.	No enhancement of oxidative stress–related atherogenic pathways beyond individual-metal exposure.
Fang et al. 2026 [67]	Cross-sectional study and prospective cohort study.	3142 patients with T2DM, 1470 patients with obstructive CAD, 1212 patients undergoing PCI	Redox-related metal mixture identified through elementomic profiling, composed of Ni, Sr, Ti, V, and Zr	Oxidative stress and redox imbalance	5-metal mixture associated with 109% higher sORP. Increased protein carbonyls, MDA, and 8-OHdG. Positive associations with obstructive CAD (OR Q4 vs. Q1 = 2.53, 95%CI: 2.05–3.12). Increased CAD extent (OR Q4 vs. Q1 = 1.88, 95%CI: 1.58–2.25). Higher risk of MACCE after PCI (HR Q4 vs. Q1 = 2.44, 95%CI 1.65–3.59). NF-κB-related cytokines mediating 9% of the association with obstructive CAD and 32% of the association with post-PCI adverse cardiovascular and cerebrovascular events.	Redox-related metal mixture-associated oxidative stress linked to atherosclerotic burden, obstructive CAD, and adverse cardiovascular outcomes
Ortega-Romero et al. 2026 [92]	Cross-sectional study	359 participants (11–19 years old)	Metal(loid) mixture (As, Cu, Mn, V).	Oxidative stress biomarkers and cardiovascular risk	Multi-biomarker oxidative stress score associated with cardiovascular risk; AOPPs (30%), MPO (18.5%), CAT (18.3%), and MGO (15.2%) as principal contributors to the oxidative stress mixture score. Metal(loid) mixture associated with increased AOPPs (β = 0.0492; 95%CI: 0.0175–0.081), MGO (β = 0.1073; 95%CI: 0.0759–0.139), MPO (β = 0.0313; 95%CI: 0.0119–0.051), and arginase (β = 0.036; 95%CI: 0.0077–0.064), with As and V as major contributors to oxidative stress alterations. No direct association between the metal(loid) mixture and cardiovascular risk.	Metal(loid) mixture-associated oxidative stress and redox imbalance linked to cardiovascular risk

Abbreviations: 8-OHdG: 8-hydroxy-2′ -deoxyguanosine; AIP: atherogenic index of plasma; AOPPs: advanced oxidation protein products; ApoE: apolipoprotein E; As: arsenic; ASCVD: atherosclerotic cardiovascular disease; As_2_O_3_: arsenic trioxide; Ba: barium; BKMR: Bayesian kernel machine regression; BPA: bisphenol A; BPAF: bisphenol AF; BPAP: bisphenol AP; BPF: bisphenol F; BPP: bisphenol P; BPS: bisphenol S; BPZ: bisphenol Z; BuP: n-butyl paraben; CAD: coronary artery disease; CAPs: concentrated ambient particulate matter; CAT: catalase; Cd: cadmium; Co: cobalt; Cr: chromium; CRI-II: Castelli Risk Index II; Cs: cesium; DHE: dihydroethidium; CVD: cardiovascular disease; Cu: copper; EPA: Environmental Protection Agency; EtP: ethylparaben; F: fluoride; HDL: high-density lipoprotein; HF: high-fat; HM: heavy metal; HO-1: heme oxygenase 1; HR: hazard ratio; HUVECs: human umbilical vein endothelial cells; IHD: ischemic heart disease; iNOS: inducible nitric oxide synthase; LF: low-fat; MACCE: major adverse cardiovascular and cerebrovascular events; MDA: malondialdehyde; MeP: methyl paraben; MGO: methylglyoxal; Mo: molybdenum; Mn: manganese; MPO: myeloperoxidase; mtDNAcn: mitochondrial DNA copy number; MVE: mixed vehicle emissions; NAC: N-acetyl-L-cysteine; NaF: sodium fluoride; NHANES: National Health and Nutrition Examination Survey; NOX: NADPH oxidase; Nqo1: NAD(P)H quinone dehydrogenase 1; NF-κB: nuclear factor kappa-light-chain-enhancer of activated B cells; Ni: nickel; Nrf2: nuclear factor erythroid 2-related factor 2; O_3_: ozone; OGG1: 8-oxoguanine DNA glycosylase; OBS: Oxidative Balance Score; OR: odds ratio; PAH: polycyclic aromatic hydrocarbon; Pb: lead; PCB: polychlorinated biphenyl; PCI: percutaneous coronary intervention; PFOS: perfluorooctane sulfonate; Q: quartile; PrP: n-propyl paraben; ROS: reactive oxygen species; SOD: superoxide dismutase; Sn: tin; Sr: strontium; sORP: static oxidation-reduction potential; TAC: total antioxidant capacity; T2DM: type 2 diabetes mellitus; TCS: triclosan; Ti: titanium; TyG: triglyceride-glucose index; V: vanadium; VOC: volatile organic compound; W: tungsten; WQS: weighted quantile sum; Zn: zinc; Zr: zirconium.

**Table 3:** Environmental mixtures and co-exposures associated with lipid dysregulation in atherosclerosis.

First Author and Year	Design	Model/Population	Exposure	Outcome	Key Findings	Relevance to Atherosclerosis
Sun et al. 2013 [48]	Experimental in vivo study	High-fructose-fed male Sprague–Dawley rats	CAPs (356 ± 52 μg/m^3^) + O_3_ (0.485 ± 0.041 ppm), 8 h/day for 9 consecutive weekdays.	Metabolic gene expression in epicardial adipose tissue	Downregulation of BAT-specific genes (*Ucp1*, *Pgc-1α*, and *Cidea*) and WAT-specific genes (*Dpt* and *Hoxc9*) in epicardial adipose tissue	Air pollution-induced adipose tissue dysfunction potentially contributing to cardiometabolic risk.
Phipps et al. 2021 [51]	Experimental in vivo study	Male C57BL/6 mice fed low-fat or high-fat diet	MVE (diesel + gasoline exhaust; 100 μg PM/m^3^), whole-body inhalation, 6 h/day, 7 days/week, for 30 days	Adipose tissue metabolic dysfunction	Increased adiposity and adipocyte hypertrophy in MVE-exposed mice. Exacerbation of adipose tissue alterations in HF-fed animals. Reduced GLUT4 and insulin receptor expression mainly in the MVE + HF group. Significant exposure–diet interaction for adipocyte hypertrophy and insulin signaling markers.	MVE-induced adipose tissue expansion, adipocyte hypertrophy, and altered metabolic signaling, particularly in the presence of a high-fat diet; potential contribution to metabolic dysregulation and atherogenesis.
Chen et al. 2025 [99]	Prospective panel study	Healthy young adults (45 participants, 180 serum samples collected across four seasonal visits),	PM_2.5_-bound metal mixture comprising 15 metals (Al, As, Se, Pb, Sb, V, Cr, Mn, Fe, Co, Ni, Cu, Zn, Mo, and Cd), assessed through personal exposure monitoring	Lipid and metabolomic dysregulation with associations to blood pressure parameters and endothelial dysfunction markers	Overall metal mixture effect negatively associated with capryloyl glycine and sphinganine; Sb as the major contributor; enrichment of sphingolipid, fatty acid, linoleic acid, glutathione, and butanoate metabolism pathways. Positive associations of Cer(d18:0/14:0), Cer(d18:0/16:0), N-palmitoylsphingosine, and arachidic acid with blood pressure parameters. Associations of lipid-related metabolites with endothelial dysfunction markers (ET-1, VEGF, ACE).	Metal mixture-associated perturbation of sphingolipid and fatty acid metabolism; ceramide-related metabolic remodeling linked to early cardiovascular injury and pathways relevant to atherogenesis.
Roth et al. 2026 [61]	Experimental in vivo study	Male Ldlr−/− mice fed an atherogenic diet	PFAS mixture (PFOA, PFOS, PFNA, PFHxS, GenX; 2 mg/L each in drinking water) for 7 weeks	Lipoprotein profile and macrophage lipid metabolism	Increased total cholesterol, IDL, LDL7, and HDL levels. Increased expression of the PPARγ-associated genes *Fabp4* and *Fasn* (fatty acid transport and synthesis) Increased *Plin1* and *Plin5* expression (lipid droplet formation and lipid storage)	PFAS mixture-induced lipoprotein remodeling, altered macrophage lipid metabolism, and foam cell formation-related pathways potentially contributing to atherogenesis.
Pan et al. 2025 [63]	Cross-sectional study (NHANES 2005–2018) integrated with network toxicology	1099 adolescents	Mixture of PFOS, PFOA, PFNA, and PFHxS assessed in serum	Dyslipidemia/lipid metabolism	Positive associations of PFAS mixture exposure with LDL-C, TC, TG, and AIP; PFOS as main contributor; PPARγ identified as a core molecular target. Identification of PPARγ signaling as a key pathway associated with PFAS-induced atherosclerosis.	PFAS mixture–associated disruption of PPAR signaling potentially contributing to lipid dysregulation and atherogenesis.
Deng et al. 2020 [64]	Experimental in vivo study	Male C57BL/6 mice	Acute co-exposure to PCB126 (0.5 mg/kg) and PFOS (250 mg/kg) by intragastric gavage; evaluation after 48 h of exposure	Lipid dysregulation (hepatic and systemic lipid metabolism)	Increased hepatic lipid accumulation. Elevated hepatic cholesterol ester levels. Altered plasma lipidome with decreased monoglycerides, diacylglycerides, phosphatidylcholines, phosphatidylinositols levels and increased Cer C16/C24 ratio. Increased Cer C16/C24 ratio. Elevated hepatic OxPLs, including PC(26:1 + 2O), PC(29:1COOH), PC(32:3 + 2O), and PC(34:1 + 2O). Oxidized phospholipids: additive interactions for PC(26:1 + 2O), PC(29:1COOH), PC(32:3 + 2O), and PC(34:1 + 2O); multiplicative interactions for PC(32:3 + 2O) and PC(34:1 + 2O)	PCB126/PFOS co-exposure-associated disruption of lipid homeostasis and pro-atherogenic remodeling
Cai et al. 2025 [101]	Nested case–control study	300 patients with type 2 diabetes mellitus (150 MACCE cases and 150 matched controls) undergoing PCI for obstructive coronary artery disease	Serum mixture of 9 PFASs (PFOA, PFUnDA, PFDA, PFOS, PFHxS, PFNA, PFBA, PFBS, and 6:2 Cl-PFESA)	2-year risk of MACCE after PCI; lipid dysregulation	PFAS mixture associated with increased MACCE risk (OR = 1.56, 95%CI: 1.27–1.92, per decile increase in WQS index). PFUnDA and PFDA as major contributors. Modest mediation by TG and LDL-C (5% of the total effect) Network analysis identifying 110 PFAS-associated lipid species, 55 of which showing significant mediation of the PFAS–MACCE association, including 26 glycerophospholipids, 13 glycerolipids, 11 sphingolipids, 3 cholesteryl esters, and 2 fatty acids.	PFAS mixture–associated perturbation of glycerophospholipid, glycerolipid, sphingolipid, and acylcarnitine metabolism potentially contributing to atherogenesis and adverse cardiovascular outcomes.
Zhang et al. 2023 [75]	Case–control study	69 IHD cases, 146 controls	Urinary mixture of bisphenols (BPA, BPF, BPS, BPAF, BPP, BPZ, BPAP), parabens (MeP, EtP, PrP, BuP), TCS, and triclocarban	Lipoprotein metabolism	0.15 mmol/L reduction in HDL per decile increase in mixture exposure. BuP and BPF as major contributors to HDL reduction. HDL mediating 91.8% of the overall mixture–IHD association. BPA-, BPF-, and BPAP-associated IHD risk partially mediated by HDL reduction (15.7%, 23.3%, and 37.7%, respectively).	Endocrine-disrupting chemical mixture-associated HDL dysregulation relevant to IHD development.
Hu et al. 2025 [102]	Cross-sectional study (NHANES 2011–2016)	2050 adults	Blood mixture of Cu, Zn, Pb, Cd, Mn, Hg, MeHg, EtHg, and IHg	Lipid dysregulation / metabolic dysfunction	Positive associations between blood concentrations of heavy metal mixtures and TyG, TyG-BMI, TyG-WC, and TyG-WHtR indices. Zn as main contributor to TyG; Cu as main contributor to TyG-BMI, TyG-WC, and TyG-WHtR. WBCs as strongest mediator of metal–TyG associations, with additional mediation by monocytes and lymphocytes.	Heavy metal mixture–associated dysregulation of glucose–lipid homeostasis and insulin resistance, potentially contributing to cardiometabolic dysfunction and atherosclerotic risk.
Zhang et al. 2024 [103]	Case–control study	116 IHD cases and 175 controls from Southern China	Mixtures of OFRs, PAEs, and polycyclic aromatic hydrocarbons (PAHs) assessed through urinary biomarkers	Glucose–lipid metabolism and IHD risk	OFR, PAE, and PAH mixtures associated with increased IHD risk: +84% (95%CI: 36–132%), +132% (95%CI: 12–252%), and +214% (95%CI: 89–331%), respectively, at the 75th percentile versus median exposure. Major contributors to mixture effects: DBP and BBOEP among OFRs, miNP among PAEs, and phenanthrene metabolites (1&9-OHPhe and 4-OHPhe) among PAHs. No significant mediation of overall mixture effects by glucose–lipid metabolism. Compound-specific mediation observed for phenanthrene metabolites through FBG, HbA1c, and TG, and for miNP through TG	OFR/PAE/PAH mixture-associated IHD risk; heterogeneous metabolic responses within mixtures; limited extrapolation of single-compound mechanisms to mixture effects

Abbreviations: OHPhe: 1- and 9-hydroxyphenanthrene; 4-OHPhe: 4-hydroxyphenanthrene; 6:2 Cl-PFESA: 6:2 chlorinated polyfluorinated ether sulfonate; ACE: angiotensin converting enzyme; AIP: atherogenic index of plasma; Al: aluminum; As: arsenic; BAT: brown adipose tissue; BBOEP: bis(2-butoxyethyl) phosphate; BPA: bisphenol A; BPAF: bisphenol AF; BPAP: bisphenol AP; BPF: bisphenol F; BPP: bisphenol P; BPS: bisphenol S; BPZ: bisphenol Z; BuP: n-butyl paraben; CAPs: concentrated ambient particulate matter; Cd: cadmium; Cer: ceramide; Co: cobalt; Cr: chromium; Cu: copper; DBP: dibutyl phosphate; EtHg: ethylmercury; ET-1: endothelin 1; FBG: fasting blood glucose; GenX: ammonium perfluoro(2-methyl-3-oxahexanoate); Fe: iron; HbA1c: hemoglobin A1c; HDL: high-density lipoprotein; HF: high-fat; Hg: mercury; IDL: intermediate-density lipoprotein; IHD: ischemic heart disease; IHg: inorganic mercury; LF: low-fat; LDL: low-density lipoprotein; LDL-C: low-density lipoprotein cholesterol; MACCE: major adverse cardiovascular and cerebrovascular events; MeHg: methylmercury; MeP: methyl paraben; miNP: monoisononyl phthalate; Mn: manganese; Mo: molybdenum; Ni: nickel; O_3_: ozone; OFR: organophosphate flame retardant; OR: odds ratio; OxPL: oxidized phospholipid; NHANES: National Health and Nutrition Examination Survey; PAE: phthalate; PAH: polycyclic aromatic hydrocarbon; Pb: lead; PC: phosphatidylcholine; PCB: polychlorinated biphenyl; PCI: percutaneous coronary intervention; PFAS: per- and polyfluoroalkyl substances; PFBA: perfluorobutanoic acid; PFBS: perfluorobutanesulfonic acid; PFDA: perfluorodecanoic acid; PFOA: perfluorooctanoic acid; PFHxS: perfluorohexanesulfonic acid; PFNA: perfluorononanoic acid; PFOS: perfluorooctane sulfonate; PFUnDA: perfluoroundecanoic acid; PPAR: peroxisome proliferator-activated receptor; PrP: n-propyl paraben; Se: selenium; Sb: antimony; TC: total cholesterol; TG: triglycerides; TyG: triglyceride-glucose index; TyG-BMI: TyG combined with Body Mass Index; TyG-WC: TyG combined with Waist Circumference; TyG-WHtR: TyG combined with Waist Height Ratio; V: vanadium; VEGF: vascular endothelial growth factor; WAT: white adipose tissue; WBC: white blood cell; WQS: weighted quantile sum; Zn: zinc.

**Table 4:** Environmental mixtures and co-exposures associated with endothelial dysfunction in atherosclerosis.

First Author and Year	Design	Model/Population	Exposure	Outcome	Key Findings	Relevance to Atherosclerosis
Rojas et al. 2022 [26]	Experimental in vivo study	Male BALB/c mice	PAH mixture (phenanthrene 55%, fluoranthene 25%, pyrene 20%); intranasal instillation (10 μL) at 10, 30, and 50 μg; 5 days/week for 5 weeks	Endothelial dysfunction markers (gene and protein expression)	Increased gene expression of ICAM-1, VCAM-1, and E-selectin. No changes in P-selectin, PECAM-1, and eNOS. Increased ICAM-1 and VCAM-1 protein expression (overall), with no significant changes in aortic tissue.	PAH-induced inflammation and endothelial activation as early mechanisms linking environmental exposure to atherosclerosis and CVD.
Phipps et al. 2021 [51]	Experimental in vivo study	Male C57BL/6 mice fed low-fat or high-fat diet	MVE (diesel + gasoline exhaust; 100 μg PM/m^3^), whole-body inhalation, 6 h/day, 7 days/week, for 30 days	Endothelial activation and vascular dysfunction	Increased vascular *ICAM-1* expression following MVE exposure irrespective of diet. *ICAM-1* expression highest in HF-fed MVE-exposed mice, with a significant exposure–diet interaction. Increased *VCAM-1* expression in MVE-exposed HF-fed mice.	MVE-induced endothelial activation and increased expression of adhesion molecules involved in leukocyte recruitment, promoting a pro-atherogenic vascular phenotype.
Quan et al. 2010 [105]	Experimental in vivo study	ApoE−/− male mice	Co-exposure to CAPs and DEG by inhalation; comparison with CAPs, DEG, and WDE exposed groups (5 h/day, 4 days/week for 5 months)	Endothelial activation, vascular dysfunction, atherosclerosis progression	Increased circulating VCAM-1 levels and enhanced phenylephrine-induced vasoconstriction following CAPs + DEG exposure. No significant interaction between CAPs and DEG for atherosclerotic plaque progression.	Air pollution co-exposure–induced endothelial activation and impaired vasomotor function; early vascular alterations relevant to atherogenesis.
Jantzen et al. 2018 [52]	Randomized double-blind crossover study	23 healthy elderly subjects	House dust (275 μg/m^3^ PM_2.5_) + O_3_ (100 ppb), 5.5 h exposure	Endothelial dysfunction and impaired vascular repair	Reduced circulating late endothelial progenitor cells (CD34^+^KDR^+^) (−48%). No significant effect following single-pollutant exposures.	House dust and O_3_ co-exposure–induced impairment of endothelial repair capacity and vascular homeostasis, potentially contributing to cardiovascular risk and atherosclerosis.
Aragon et al. 2016 [54]	In vivo inhalation exposure with subsequent in vitro/ex vivo serum bioactivity assays	C57BL/6 mice	Acute inhalation exposure (6 h) to road dust (349 μg/m^3^ PM_2.5_), road dust + O_3_ (344 μg/m^3^ PM_2.5_ + 0.33 ppm O_3_), road dust + MVE; 342 μg/m^3^ PM_2.5_, MVE gases, MVE particulate matter (MVE-PM; 328 μg/m^3^ PM_2.5_), or wood smoke (380 μg/m^3^ PM_2.5_); serum collected 18–24 h post-exposure and incubated with murine cerebrovascular endothelial cells for 4 h.	Endothelial activation and vascular dysfunction	Road dust + O_3_: increased ICAM-1 and VCAM-1 expression. Road dust + MVE, MVE-PM, and MVE gases: ~20–40% reduction in acetylcholine-mediated vasorelaxation. Increased serum-induced vasoconstriction following road dust + MVE exposure.	Air pollution mixture-associated endothelial activation and impaired vascular reactivity.
Zhang et al. 2016 [81]	Experimental in vitro study	H9C2 rat cardiomyocytes	Heavy metal-containing PM_2.5_ mixture (Cr, Ni, Cu, Cd, Pb, Zn, Mn, and Co) collected during different seasons in a coal-burning region of northern China; 0–10 μg/mL for 24 h	Endothelial activation	Dose-dependent ICAM-1 upregulation; 1.78-fold increase following spring PM_2.5_ exposure and 2.24-fold increase following winter PM_2.5_ exposure.	PM_2.5_-associated metal mixture-induced endothelial activation and vascular injury relevant to atherogenesis
McGraw et al. 2021 [107]	Cross-sectional study	Nonsmokers with moderate-to-high CVD risk (n = 346; endothelial function subset n = 70)	Urinary VOC mixture including acrolein, 1,3-butadiene, and crotonaldehyde metabolites	Endothelial dysfunction and vascular function	Reduced RHI associated with 3HPMA (−4.4%, 95%CI: −7.2 to −0.0) and DHBMA (−3.9%, 95%CI: −9.4 to −0.0). 3HPMA-associated increase in SBP (+0.98 mmHg, 95%CI 0.06–1.91). BKMR identification of 3HPMA as the principal contributor to vascular dysfunction within the VOC mixture.	VOC mixture-associated endothelial dysfunction and impaired vascular homeostasis
He et al. 2022 [30]	Experimental in vitro study	Primary HUVECs	Mixture of 16 EPA priority PAHs; exposure concentrations derived from blood levels reported in the Chinese general population and tested at environmental (1×) and higher doses (10×–1000×); 48 h treatment duration	Endothelial cell migration, wound healing, tube formation, morphology, oxidative stress, inflammation, and apoptosis	Impaired endothelial migration (wound-healing and transwell assays) and tube formation. Elongated, fibroblast-like endothelial phenotype. Reduced angiogenic and chemotactic capacity.	PAH mixture–induced endothelial dysfunction; impaired angiogenic capacity and endothelial repair; promotion of a pro-atherogenic endothelial phenotype
Moreno-Gómez-Toledano et al. 2023 [108]	NHANES-based cross-sectional study and experimental in vitro study	3014 adults (313 with heart disease; 2701 healthy controls); murine aortic endothelial cells	Bisphenol mixture (BPA, BPF, and BPS)	Endothelial dysfunction and endothelial cell injury	Positive association between urinary bisphenol mixture exposure and heart disease (OR = 1.20, 95%CI: 1.05–1.38). Greater reduction in endothelial cell viability following bisphenol mixture exposure compared with BPA alone. Increased Annexin V-positive cells. Increased caspase-3 and caspase-8 expression. No changes in RIP3 or MLKL expression, indicating apoptosis rather than necroptosis.	Bisphenol mixture-associated endothelial injury and apoptosis relevant to vascular dysfunction and atherogenesis
Ma et al. 2017 [90]	Experimental in vitro study	Primary HUVECs	As_2_O_3_, 5 μM and NaF, 1 mM, alone and in combination for 24 h	Endothelial dysfunction / endothelial activation	Increased endothelial apoptosis (19.2% after As exposure, 18.8% after F exposure, and 35.9% after co-exposure vs. 5.0% in controls). Upregulation of *VCAM-1*, *ICAM-1*, and *PTX3* expressions. Reduced NO production. Less pronounced increases in VCAM-1, ICAM-1, and PTX3 expression following co-exposure than after F exposure	Mixture-specific endothelial effects of As-F co-exposure, with evidence of both vascular injury and antagonistic interactions.
Ma et al. 2012 [68]	Experimental in vivo study	New Zealand White rabbits	As (13 mg/L As_2_O_3_), F (50 mg/L NaF), or combined As + F exposure via drinking water; 6-month exposure	Endothelial activation	Increased VCAM-1 and P-selectin expression at mRNA and protein levels following As, F, and As + F exposure. Reduced NO production and increased endothelial apoptosis under co-exposure. Lower induction of VCAM-1 and P-selectin under co-exposure compared with either contaminant alone	As–F co-exposure–associated endothelial activation relevant to early atherogenesis; evidence of non-additive interactions during co-exposure.
Subramaniam et al. 2024 [71]	Combined in vitro and in vivo study	RAW264.7 macrophages, C166 endothelial cells, and ApoE−/− mice	Environmentally relevant low-dose As/Cd mixture (5–50 ppb As; 1.5–5 ppb Cd) in drinking water for 13 weeks in male and female ApoE−/− mice; parallel low-dose in vitro exposures	Endothelial dysfunction; aortic arch plaque burden; plaque composition	No significant changes in VCAM-1 expression following As, Cd, or combined exposure. No significant increase in aortic arch plaque burden in either sex following co-exposure. Increased lesion size with Cd alone in males and As alone in females. Limited alterations in plaque composition, including macrophage accumulation, smooth muscle cell content, collagen deposition, and necrotic core formation	Absence of endothelial activation, plaque progression, and adverse plaque remodeling beyond individual-metal exposure.
Gong et al. 2025 [46]	Cross-sectional study (NHANES 2005–2018) integrated with network toxicology	12,127 US adults	Urinary concentrations of 10 DEHP metabolites	Prevalent CVD; network toxicology pathways	Positive association between phthalate mixture exposure and CVD (OR = 1.21, 95%CI: 1.07–1.37) (WQS). Identification of PIK3CA as a core target. Enrichment of the PI3K-Akt signaling pathway.	Phthalate mixture–associated perturbation of PI3K-Akt pathway signaling, potentially contributing to endothelial dysfunction and vascular injury.
Wu et al. 2025 [14]	Cross-sectional study integrating epidemiological analyses and toxicogenomic bioinformatics	2291 Chinese participants from rural areas	Mixed pesticide exposure (34 selected pesticides including neonicotinoids, organochlorine pesticides, organophosphorus pesticides, pyrethroids, and herbicides measured in blood and urine).	10-year ASCVD risk, endothelial dysfunction, and apoptosis-related pathways associated with ASCVD.	Total pesticide mixture exposure positively associated with 10-year ASCVD risk in QGC (OR = 3.223, 95%CI: 2.196–4.730) and WQS models (OR = 4.642, 95%CI: 3.070–7.020). Linear dose–response relationship between overall pesticide mixture exposure and high ASCVD risk. Identification of 112 pesticide-related atherosclerosis target genes; enrichment of PI3K–Akt signaling and apoptosis pathways; AKT1, TP53, BCL2, CASP3, and CASP9 identified among the major hub genes linking pesticide exposure to ASCVD.	Pesticide mixture-associated dysregulation of PI3K–Akt signaling involved in endothelial cell survival and vascular homeostasis, together with altered apoptotic pathways potentially contributing to vascular injury and atherogenesis.
Pan et al. 2025 [63]	Cross-sectional study (NHANES 2005–2018) integrated with network toxicology	1099 adolescents	Mixture of PFOS, PFOA, PFNA, and PFHxS assessed in serum	Endothelial dysfunction / vascular remodeling	Positive associations of PFAS mixture exposure with AIP (WQS β = 0.03, 95%CI 0.006–0.04); PFOA identified as the main contributor. CA2 identified as a potential molecular target of PFAS-associated atherosclerosis.	PFAS mixture–associated vascular remodeling and structural vascular alterations potentially contributing to atherosclerosis progression.
Deng et al. 2020 [64]	Experimental in vivo study	Male C57BL/6 mice	Acute co-exposure to PCB126 (0.5 mg/kg) and PFOS (250 mg/kg) by intragastric gavage; evaluation after 48 h of exposure	Endothelial dysfunction and vascular injury biomarkers	Increased hepatic ICAM-1 and PAI-1 expression. Elevated circulating PAI-1 levels. Additive synergistic interaction between PFOS and PCB126 for PAI-1 expression. No effects on *Sele* expression.	Synergistic PFOS/PCB126 mixture-induced activation of endothelial dysfunction and thrombosis pathways relevant to atherosclerosis.

Abbreviations: ApoE: apolipoprotein E; 3HPMA: N-Acetyl-S-(3-hydroxypropyl)-L-cysteine; AIP: atherogenic index of plasma; ASCVD: atherosclerotic cardiovascular disease; As: arsenic; As_2_O_3_: arsenic trioxide; BPA: bisphenol A; BPF: bisphenol F; BPS: bisphenol S; CA2: carbonic anhydrase 2; CAPs: concentrated ambient particulate matter; CVD: cardiovascular disease; DEG: diesel exhaust gases; DHBMA: N-Acetyl-S-(3,4-dihydroxybutyl)-L-cysteine; eNOS: endothelial nitric oxide synthase; DEHP: di-2-ethylhexyl phthalate; EPA: Environmental Protection Agency; HF: high-fat; HUVECs: human umbilical vein endothelial cells; ICAM-1: intercellular adhesion molecule 1; LF: low-fat; MLKL: mixed lineage kinase domain-like pseudokinase; MVE: mixed vehicle emissions; NaF: sodium fluoride; NHANES: National Health and Nutrition Examination Survey; NO: nitric oxide; O_3_: ozone; OR: odds ratio; PAH: polycyclic aromatic hydrocarbon; PAI-1: plasminogen activator inhibitor-1; PCB: polychlorinated biphenyl; PECAM-1: platelet endothelial cell adhesion molecule 1; PFOA: perfluorooctanoic acid; PFHxS: perfluorohexanesulfonic acid; PFNA: perfluorononanoic acid; PFOS: perfluorooctane sulfonate; PI3K–Akt: phosphoinositide 3-kinase/protein kinase B; PM: particulate matter; PTX3: pentraxin 3; QGC: quantile-based *g*-computation; RHI: reactive hyperemia index; RIP3: receptor-interacting serine/threonine kinase 3; SBP: systolic blood pressure; Sele: E-selectin; VCAM-1: vascular cell adhesion molecule 1; VOC: volatile organic compound; WDE: whole diesel exhaust; WQS: weighted quantile sum.

**Table 5:** Environmental mixtures and co-exposures associated with epigenetic alterations in atherosclerosis.

First Author and Year	Design	Model/Population	Exposure	Outcome	Key Findings	Relevance to Atherosclerosis
Lin et al. 2020 [119]	Cross-sectional study	738 Taiwanese adolescents and young adults	Urinary Pb and Cd concentration	Global DNA methylation (5 mdC/dG) and CIMT	In separate analyses, both urinary Pb and Cd concentrations positively associated with 5 mdC/dG (*p* < 0.001) and CIMT (*p* < 0.001). In the co-exposure models, Pb associated with 5 mdC/dG (β = 0.47, *p* < 0.001) and CIMT (β = 11.41, *p* < 0.001); Cd associated only with CIMT (β = 5.47, *p* = 0.027). Higher odds of CIMT >75th percentile with increasing Pb levels among participants with 5 mdC/dG above the median (OR = 1.67, 95%CI: 1.17–2.46). Pb associated with CIMT both directly and indirectly through global DNA methylation; Cd associated directly with CIMT (SEM).	Pb/Cd co-exposure associated with increased CIMT; global DNA methylation identified as a potential epigenetic mediator of Pb-related subclinical atherosclerosis.
Li et al. 2025 [123]	Prospective cohort study	7622 Chinese participants aged 45 years and older	Residential ambient exposure to a multi-pollutant air mixture (PM_2.5_, PM_10_, NO_2_, SO_2_)	DNA methylation and CVD risk	PM_2.5_, PM_10_, NO_2_, and SO_2_ individually associated with increased risk of CVD by 6.2%, 4.4%, 9.3%, and 6.1%, respectively, with a linear dose–response relationship. Mixed exposure to PM_2.5_, PM_10_, NO_2_, and SO_2_ associated with increased CVD risk (WQS OR = 1.010, 95%CI: 1.002–1.018), with PM_10_ (61%) and PM_2.5_ (20%) as the major contributors. Genetically predicted methylation at PM_2.5_-related cg01065697 (*PRDM16*) associated with MI (OR = 1.287, 95%CI: 1.162–1.425) and IHD (OR = 1.258, 95%CI: 1.136–1.394). NO_2_-related cg07091220 (*ZNF827*) associated with MI (OR = 1.243, 95%CI: 1.129–1.369), while cg15474579 (*CDKN1A*), cg16348358 (*LCK*), and cg19869422 (*SMG6*) associated with HF risk.	Air pollution mixture-associated cardiovascular risk; *PRDM16*-, *ZNF827*-, *CDKN1A*-, *LCK-*, and *SMG6*-related DNA methylation pathway
Wahlang et al. 2016 [128]	Experimental in vitro study	Primary HUVECs	PCB mixture (Aroclor 1260, 10 μM, 16 h)	Endothelial miRNA expression	Altered expression of 557 miRNAs; 21 miRNAs related to vascular diseases. Increased miR-21, miR-31, miR-126, miR-221, miR-222.	PCB-induced miRNA dysregulation; links to vascular inflammation and atherogenesis initiation.
Shan et al. 2020 [133]	Experimental in vivo study	Male ApoE−/− mice	Co-exposure to TCDD (15 μg/kg bw) and Aroclor 1254 (55 mg/kg bw) by intraperitoneal injection, administered four times over a 6-week period (two injections during week 1 and two during week 4, 3 days apart)	miRNA dysregulation and epigenetic remodeling	68 differentially expressed miRNAs and 1312 differentially expressed mRNAs. 2.31-fold increase in atherosclerotic lesion area. Upregulation of mRNA levels of *MMP12* (11.45-fold), *MMP13* (63.83-fold), *CD36* (5.25-fold), and *ICAM-1* (2.25-fold). Dysregulation of miR-26a-5p, miR-193a-3p, miR-30c-5p, miR-130a-3p, and miR-376a-3p within cardiovascular and atherosclerosis-related networks.	TCDD/Aroclor 1254 mixture-associated miRNA-mediated regulation of cardiovascular development and atherosclerosis signaling pathways
Zhang et al. 2024 [134]	Longitudinal panel study (repeated measures across 3 seasons)	123 middle-aged community adults (China); repeated urine and plasma measurements	Urinary PAH metabolites (10 OH-PAHs, creatinine-adjusted); repeated daily measurements Lag structure (lag 0–3 days) to assess short-term exposure effects; strongest associations observed at lag 0.	Plasma arterial stiffness-related miRNAs (miR-146a, miR-222, others)	Positive associations of PAH mixture (lag 0) with miR-146a and miR-222 (BKMR); 9-OHFlu as the dominant contributor (highest PIP). IQR increase in urinary 9-OHFlu associated with increases in miR-146a of 0.23–0.34 (95%CI: 0.02–0.54) and in miR-222 of 0.16–0.17 (95%CI: 0.02–0.32), depending on background mixture levels. At lag 0, 9-OHFlu, 2-OHPh, and 9-OHPh positively associated with miR-146a (+10.4% to +23.6%), while 9-OHFlu associated with higher miR-222 levels (+16.0%; 95%CI: 5.9–27.0%)	PAH mixture–induced miRNA dysregulation; links to inflammation, endothelial dysfunction, and arterial stiffness; early epigenetic mechanisms of atherogenesis.
Bai et al. 2022 [136]	Longitudinal panel study (repeated measures across 3 seasons)	123 middle-aged adults (China); 338 paired urine–blood samples	Urinary concentrations of 10 phthalate metabolites (creatinine-adjusted)	Plasma miRNA expression (miR-146a, miR-125b, miR-222, miR-126, miR-21); arterial stiffness assessed by ABI and baPWV	Positive association between phthalate metabolite mixture and miR-146a, miR-125b, and miR-222 (WQS index: +11.85%, +10.05%, and +9.20%, respectively); MMP and MBP identified as the main drivers of the mixture effect. Dose–response associations between individual phthalate metabolites (including MMP, MBP, and MiBP) and increased plasma miRNA levels. miR-146a mediation of the association between MMP and MiBP and ABI (31.6% and 21.3%, respectively). Inverse association between miR-146a and ABI.	Phthalate mixture–induced miRNA dysregulation; links to vascular calcification, inflammation, and endothelial dysfunction; miRNA-mediated epigenetic mechanisms of arterial stiffness and early atherogenesis.

Abbreviations: 2-OHPh: 2-hydroxyphenanthrene; 5 mdC/dG: 5-methyl-2′-deoxycytidine/deoxyguanosine ratio; ABI: ankle-brachial index; baPWV: brachial-ankle pulse wave velocity; BKMR: Bayesian kernel machine regression; Cd: cadmium; CDKN1A: cyclin-dependent kinase inhibitor 1A; CIMT: carotid intima media thickness; CVD: cardiovascular disease; DEHP: di-2-ethylhexyl phthalate; HF: heart failure; HUVECs: human umbilical vein endothelial cells; ICAM-1: intercellular adhesion molecule 1; IHD: ischemic heart disease; LCK: lymphocyte-specific protein tyrosine kinase; MI: myocardial infarction; miRNA: microRNA; MBP: mono-n-butyl phthalate; MiBP: mono-iso-butyl phthalate; MMP: mono-methyl phthalate; MMP12: metalloproteinase 12; MMP13: metalloproteinase 13; NHANES: National Health and Nutrition Examination Survey; NO_2_: nitrogen dioxide; PAH: polycyclic aromatic hydrocarbon; PCB: polychlorinated biphenyl; PIP: posterior inclusion probabilities; PRDM16: PR/SET domain 16; OH-PAH: monohydroxylated polycyclic aromatic hydrocarbon; OR: odds ratio; Pb: lead; PM: particulate matter; SEM: structural equation modeling; SMG6: SMG6 nonsense mediated mRNA decay factor; SO_2_: sulfur dioxide; WQS: weighted quantile sum; ZNF827: zinc finger protein 827.

## Data Availability

No new data were created or analyzed in this study. Data sharing is not applicable to this article.

## Supplementary Materials

The following supporting information can be downloaded at https://www.mdpi.com/article/10.3390/ijms27177624/s1.

## Abbreviations

The following abbreviations are used in this manuscript:

8-OHdG	8-hydroxy-2′-deoxyguanosine
AhR	Aryl hydrocarbon receptor
ASCVD	Atherosclerotic cardiovascular disease
BKMR	Bayesian kernel machine regression
BPA	Bisphenol A
CAPs	Concentrated ambient particulate matter
CIMT	Carotid intima-media thickness
CVD	Cardiovascular disease
EDC	Endocrine disrupting chemical
HDL	High-density lipoprotein
IHD	Ischemic heart disease
IL	Interleukin
LDL	Low-density lipoprotein
MCP-1	Macrophage/monocyte chemoattractant protein-1
MI	Myocardial infarction
NF-κB	Nuclear factor kappa-light-chain-enhancer of activated B cells
NO	Nitric oxide
Nrf2	Nuclear factor erythroid 2-related factor 2
OBS	Oxidative Balance Score
OxPL	Oxidized phospholipid
PAH	Polycyclic aromatic hydrocarbon
PCB	Polychlorinated biphenyl
PFAS	Per- and polyfluoroalkyl substances
PFOS	Perfluorooctane sulfonic acid
POP	Persistent organic pollutant
PM	Particulate matter
RAS	Renin–angiotensin system
ROS	Reactive oxygen species
THM	Trihalomethane
TNF-α	Tumor necrosis factor alpha
TyG	Triglyceride–glucose index
VOC	Volatile organic compound
WQS	Weighted quantile sum
