# Supplementary material for: A Perfect Storm of Pollutants: Environmental Mixtures in the Pathogenesis of Atherosclerosis
Source: Int J Mol Sci. 2026 Aug 25;27(17):7624. doi: 10.3390/ijms27177624 (PMC13566070; doi:10.3390/ijms27177624)
Supplement: Supplementary file 1 [file ijms-27-07624-s001.zip › ijms-4494336-supplementary.pdf]

Supplementary Material S1. Search Strategies Used in PubMed

PubMed

("Atherosclerosis"[Mesh] OR atheroscleros* OR atherogenesis OR "atherosclerotic plaque*" OR ASCVD OR "atherosclerotic cardiovascular disease" OR "coronary heart disease" OR "coronary artery disease" OR "myocardial infarction" OR angina OR "ischemic stroke" OR "vascular disease*" OR "cardiovascular disease*" OR "carotid intima-media thickness" OR "arterial stiffness")

AND

("environmental mixture*" OR "chemical mixture*" OR mixture* OR coexposure* OR "co-exposure*" OR multipollutant* OR "multiple pollutant*" OR exposom* OR "cumulative exposure*" OR "combined exposure*" OR "mixed exposure*")

AND

("Air Pollution"[Mesh] OR PM2.5 OR PM10 OR "particulate matter" OR metal* OR "heavy metal*" OR lead OR cadmium OR arsenic OR mercury OR fluoride OR pesticide* OR "Pesticides"[Mesh] OR herbicide* OR insecticide* OR dinitroaniline OR trifluralin OR pendimethalin OR PAH OR "polycyclic aromatic hydrocarbon*" OR "endocrine disrupting chemical*" OR EDC OR "persistent organic pollutant*" OR POP OR POPs OR PCB OR "polychlorinated biphenyl*" OR phthalate* OR bisphenol* OR PFAS OR PFOA OR PFOS OR "perfluoroalkyl*" OR "polyfluoroalkyl substance*" OR "fluorocarbons*" OR microplastic* OR nanoplastic* OR "risk factors")

AND

("oxidative stress" OR inflammation OR "inflammatory response*" OR "endothelial dysfunction" OR autophagy OR genotoxicity OR "DNA damage" OR epigenetic* OR methylation OR "DNA methylation" OR miRNA OR microRNA OR "gene expression" OR "mitochondrial dysfunction" OR "mitochondrial DNA" OR "mtDNA copy number" OR "foam cell*" OR "endothelial cell*" OR macrophage* OR "lipid accumulation" OR "lipid metabolism" OR "cholesterol efflux" OR lipoprotein*)
